# Acute effects of the imidacloprid metabolite desnitro-imidacloprid on human nACh receptors relevant for neuronal signaling

**DOI:** 10.1007/s00204-021-03168-z

**Published:** 2021-10-10

**Authors:** Dominik Loser, Karin Grillberger, Maria G. Hinojosa, Jonathan Blum, Yves Haufe, Timm Danker, Ylva Johansson, Clemens Möller, Annette Nicke, Susanne H. Bennekou, Iain Gardner, Caroline Bauch, Paul Walker, Anna Forsby, Gerhard F. Ecker, Udo Kraushaar, Marcel Leist

**Affiliations:** 1 NMI Natural and Medical Sciences Institute at the University of Tübingen, 72770 Reutlingen, Germany; 2 In Vitro Toxicology and Biomedicine, Department Inaugurated by the Doerenkamp-Zbinden Foundation, University of Konstanz, 78457 Konstanz, Germany; 3 Department of Pharmaceutical Chemistry, University of Vienna, Vienna, Austria; 4 Department of Biochemistry and Biophysics, Stockholm University, 106 91 Stockholm, Sweden; 5 Walther Straub Institute of Pharmacology and Toxicology, Faculty of Medicine, LMU Munich, 80336 Munich, Germany; 6 Life Sciences Faculty, Albstadt-Sigmaringen University, 72488 Sigmaringen, Germany; 7 Technical University of Denmark, Kongens Lyngby, Denmark; 8 CERTARA UK Limited, Simcyp Division, Level 2-Acero, 1 Concourse Way, Sheffield, S1 2BJ UK; 9 Cyprotex Discovery Ltd, No. 24 Mereside, Alderley Park, Cheshire, SK10 4TG UK

**Keywords:** Live-cell calcium imaging, Pesticide metabolism, Nicotine, Developmental neurotoxicity, Molecular docking, Oocyte recording

## Abstract

Several neonicotinoids have recently been shown to activate the nicotinic acetylcholine receptor (nAChR) on human neurons. Moreover, imidacloprid (IMI) and other members of this pesticide family form a set of diverse metabolites within crops. Among these, desnitro-imidacloprid (DN-IMI) is of special toxicological interest, as there is evidence (i) for human dietary exposure to this metabolite, (ii) and that DN-IMI is a strong trigger of mammalian nicotinic responses. We set out here to quantify responses of human nAChRs to DN-IMI and an alternative metabolite, IMI-olefin. To evaluate toxicological hazards, these data were then compared to those of IMI and nicotine. Ca^2+^-imaging experiments on human neurons showed that DN-IMI exhibits an agonistic effect on nAChRs at sub-micromolar concentrations (equipotent with nicotine) while IMI-olefin activated the receptors less potently (in a similar range as IMI). Direct experimental data on the interaction with defined receptor subtypes were obtained by heterologous expression of various human nAChR subtypes in *Xenopus laevis* oocytes and measurement of the transmembrane currents evoked by exposure to putative ligands. DN-IMI acted on the physiologically important human nAChR subtypes α7, α3β4, and α4β2 (high-sensitivity variant) with similar potency as nicotine. IMI and IMI-olefin were confirmed as nAChR agonists, although with 2–3 orders of magnitude lower potency. Molecular docking studies, using receptor models for the α7 and α4β2 nAChR subtypes supported an activity of DN-IMI similar to that of nicotine. In summary, these data suggest that DN-IMI functionally affects human neurons similar to the well-established neurotoxicant nicotine by triggering α7 and several non-α7 nAChRs.

**Supplementary Information:**

The online version contains supplementary material available at 10.1007/s00204-021-03168-z.

## Introduction

The toxicological assessment of many pesticides is complicated by the fact that there is not only exposure to the original substances, but also to their many metabolites formed in the environment. This also applies to the neonicotinoids, a class of insecticides with long persistence within crops (Simon-Delso et al. 2015; Craddock et al. 2019; Thompson et al. 2020). They comprise, e.g., imidacloprid (IMI), acetamiprid, clothianidin, and thiacloprid. With a global market turnover of > 1 billion € (Jeschke et al. 2011; Sparks and Nauen 2015), this group of compounds has dominated many pesticide application domains and thus has led to widespread human exposure (Klarich et al. 2017; Craddock et al. 2019; Thompson et al. 2020). The neonicotinoids had a worldwide market share of the insecticide sales of around 25% in 2014–2018 (Bass et al. 2015; Casida 2018; Sparks et al. 2020). IMI accounted for around one-third of neonicotinoid use (Bass et al. 2015). In the US, the use of IMI for crop protection was estimated to be roughly around 1000 tons per year from 2011 to 2014 (Douglas and Tooker 2015; Craddock et al. 2019; US Geological Survey 2021). The insecticidal mode of action is based on the over-activation of the nicotinic acetylcholine receptor (nAChR) of the target species. This activity has been assumed to be relatively specific for the insect nervous system (Brown et al. 2006; Tan et al. 2007), as neonicotinoids have been developed to exhibit a higher affinity for insect nAChRs compared to vertebrate paralogs (Tomizawa et al. 2000; Tomizawa and Casida 2005; Casida 2018). However, some studies suggest adverse effects of neonicotinoids on mammals (Abou-Donia et al. 2008; Duzguner and Erdogan 2012; Burke et al. 2018; Berheim et al. 2019). A broad toxicological debate has been triggered by the observation that acetamiprid and IMI activated the nAChRs on neonatal rat neurons in the low µM range (Kimura-Kuroda et al. 2012). The relevance of this finding for human toxicology is further supported by a recent study using cultured human neurons. Clear nAChR signaling and also pronounced receptor desensitization were demonstrated for several neonicotinoids at concentrations that may be reached by dietary or accidental exposure (Loser et al. 2021a).

Food products intended for human consumption have high detection rates for IMI (Chen et al. 2014; Craddock et al. 2019; Thompson et al. 2020). In addition, several metabolites are found. The transformation of the parent compounds can arise via abiotic (photolysis, hydrolysis, and chlorination) or biological (microbial, fungal, and plant) processes (Simon-Delso et al. 2015; Thompson et al. 2020). One important metabolic step is the reduction of the nitro group of IMI to form aminoguanidine derivatives or derivatives that entirely lack the nitro group (e.g., DN-IMI) (Ford and Casida 2006). Besides cytochrome P450 enzymes, especially aldehyde oxidase seems to play an important role in this biotransformation (Schulz-Jander and Casida 2002; Schulz-Jander et al. 2002; Dick et al. 2005; Swenson and Casida 2013; Simon-Delso et al. 2015; Vardavas et al. 2018).

Imidacloprid-olefin (IMI-olefin) has been detected in honey (Codling et al. 2016; Thompson et al. 2020), and DN-IMI is a major IMI degradation product in the environment (Anon 2006; Koshlukova 2006). The latter metabolite is produced abiotically by photodegradation (17% of all IMI), but also biotically as the dominant bacterial metabolite, and as a major metabolite in many plants (Anon 2006; Koshlukova 2006). It has, e.g., been found in drinking water (Klarich Wong et al. 2019; Wan et al. 2020) and it has been reported to be formed in diverse foods such as apples, tomatoes, eggplants, and potatoes, where it accounted for around 10–30% of IMI degradation products. It reached concentrations in the 10–30 µg/kg range in apples and potatoes and up to 300 µg/kg in fodder corn (Anon 2006). The outdoor use of IMI has been banned in Europe in 2018 (European Commission 2018), due to unacceptable toxicity risks for bees (European Food Safety Authority [EFSA] 2016), and in 2020 the approval of IMI expired (European Commission 2020). However, exposure via the diet still occurs via imported food commodities, and until now, several EU countries still grant temporary exemptions and notify the EU of these emergency authorizations (https://ec.europa.eu/food/plant/pesticides/eu-pesticides-database/ppp/pppeas/screen/home). The current dietary risk assessment on IMI in Europe covers the exposure to the parent compound IMI and its metabolites. However, there is no specific residue definition for DN-IMI (European Food Safety Authority [EFSA] et al. 2019). This means that it is assumed that the toxicological potency is similar to the parent compound.

DN-IMI has also been detected in mice exposed to IMI. This suggests that it can also be produced within mammals by endogenous metabolism (Ford and Casida 2006; Swenson and Casida 2013). This is consistent with findings of DN-IMI and IMI-olefin in human urine samples analyzed in a recent biomonitoring study (Wang et al. 2020).

The previous knowledge of IMI metabolism shows that a shift in the bioactivity spectrum can occur. For instance, the metabolite DN-IMI has a strongly reduced potency on insect nAChRs, but in turn an increased affinity for mammalian nAChRs (Liu et al. 1993; Chao and Casida 1997; Tomizawa et al. 2000). This is in line with studies in mice that suggested a higher toxicity of DN-IMI, compared to its parent compound IMI (Chao and Casida 1997; Tomizawa et al. 2000). Furthermore, binding assays using mammalian nAChRs have shown that DN-IMI has an affinity similar to the high-affinity ligand nicotine (Tomizawa and Casida 1999; D’Amour and Casida 1999; Tomizawa et al. 2000). Nicotine is a well-known neurotoxicant and developmental neurotoxicant for vertebrates, including man (Levin et al. 1993; Slikker Jr et al. 2005; LeSage et al. 2006; Grandjean and Landrigan 2006; Dwyer et al. 2009; Slotkin et al. 2016; Zahedi et al. 2019). Therefore, IMI metabolites mimicking the activity profile of nicotine on human receptors are of high toxicological concern.

The activation of ionotropic receptors like nAChRs on neurons leads to a depolarization of the cell membrane, and thereby, activates voltage-dependent Ca^2+^ channels. The transient influx of Ca^2+^ into the cell increases the intracellular free Ca^2+^ concentration ([Ca^2+^]_i_), which can be measured by Ca^2+^-imaging in neuronal cell cultures (Leist and Nicotera 1998; Sirenko et al. 2019; Grunwald et al. 2019; Loser et al. 2021b). This method is based on the quantifications of fluorescence signals of calcium-sensitive dyes introduced into the cells, and it is amenable to high-throughput formats (Sirenko et al. 2019; Karreman et al. 2020; Brüll et al. 2020; Loser et al. 2021b). Alternatively, xenobiotic effects on individual nAChR subtypes may be measured directly by the recording of the transmembrane currents in *Xenopus laevis* oocytes that heterologously express human receptors of interest. The basis of this method is the injection of mRNA coding for human neurotransmitter receptor subunits into the cells. It is well known that this experimental system has a high efficiency for protein translation and functional insertion of the respective receptors in the cell membrane. The large size of the oocytes allows the current flow through the cell membrane (triggered by agonists) to be measured by two sharp microelectrodes placed inside the cell. The test method obtains its specificity from the strong heterologous expression of the respective receptor. (Bermudez and Moroni 2006; Moroni et al. 2006; Jonsson et al. 2006; Carbone et al. 2009; Mineur et al. 2009; Mazzaferro et al. 2011; Harpsøe et al. 2011; Li et al. 2011; Benallegue et al. 2013).

The human neuronal precursor cell line LUHMES and the neuroblastoma cell line SH-SY5Y can be differentiated into post-mitotic neurons (Lopes et al. 2010; Scholz et al. 2011), and they are often used as a model system to investigate adverse effects on human neurons (Tomizawa and Casida 1999; Gustafsson et al. 2010; Krug et al. 2013, 2014; Zhang et al. 2014; Ring et al. 2015; Lohren et al. 2015; Attoff et al. 2016, 2020; Smirnova et al. 2016; Harris et al. 2017; Tong et al. 2017; Witt et al. 2017; Delp et al. 2018a, b, 2019; Brüll et al. 2020). The utility of these cell models for functional neurotoxicity testing has been demonstrated for agents that affect voltage-dependent sodium channels or ionotropic receptors (Loser et al. 2021a, b). Both cell types express functional nAChRs and have been used in Ca^2+^-imaging assays to study the effects of several neonicotinoids (Loser et al. 2021a).

In this study, we explored whether DN-IMI possesses a potential neurotoxicity or developmental neurotoxicity hazard, by acting on nAChRs of human neurons. The IMI metabolite was chosen for this study, as it may be directly ingested by food. However, it is also relevant as it may be generated in individuals exposed to IMI. We compared the signaling effects of DN-IMI on LUHMES neurons and SH-SY5Y to that of IMI and nicotine. To determine differences in nAChR subtype selectivity of the compounds, we further investigated the agonist activity of these compounds on human α4β2, α7, and α3β4 nAChR subtypes, expressed in *Xenopus laevis* oocytes, and we developed a molecular docking approach explaining these findings. To gather background information on the persistence and distribution of DN-IMI in man, a toxicokinetic model was implemented and parameterized by metabolism data from human hepatocytes. The broad data set of this study was used for a preliminary risk assessment of DN-IMI.

## Materials and methods

### Materials and chemicals

An overview of experimental tool compounds and toxicants is given in Table S1. Consumables are indicated in the specific methods paragraphs. Chemical structures of imidacloprid (IMI) (https://pubchem.ncbi.nlm.nih.gov/compound/86287518#section=2D-Structure), desnitro-imidacloprid (DN-IMI) (https://pubchem.ncbi.nlm.nih.gov/compound/10130527#section=2D-Structure) and imidacloprid-olefin (IMI-olefin) (https://pubchem.ncbi.nlm.nih.gov/compound/14626249#section=2D-Structure) were obtained from PubChem and visualized in ChemDraw JS (version 19.0.0-CDJS-19.0.x.9 + da9bec968, PerkinElmer).

### LUHMES cell culture

The cultivation of the LUHMES cells was performed as described earlier (Scholz et al. 2011; Krug et al. 2013; Schildknecht et al. 2013). In brief, LUHMES cells were cultured in standard cell culture flasks (Sarstedt) that were pre-coated with 50 µg/ml poly-l-ornithine (PLO) and 1 µg/ml fibronectin (Sigma Aldrich) in H_2_O overnight at 37 °C. The cells were maintained in proliferation medium containing advanced DMEM/F12 (Gibco) with 2 mM l-glutamine (Sigma Aldrich), 1 × N2-supplement (Gibco), and 40 ng/ml recombinant human basic fibroblast growth factor (FGF-2, R&D Systems). The cells were kept at 37 °C and 5% CO_2_ and passaged three times a week when the culture reached a confluency of 75–90%. Cells were used until passage 18. For differentiation, cells were cultured in differentiation medium consisting of advanced DMEM/F12 (Gibco) supplemented with 2 mM l-glutamine (Sigma Aldrich), 1 × N2-supplement (Gibco), 1 mM N6,2′-0-dibutyryl 3′,5′-cyclic adenosine monophosphate (cAMP) (Sigma Aldrich), 1 µg/ml tetracycline (Sigma Aldrich) and 2 ng/ml recombinant human glial cell-derived neurotrophic factor (GDNF, R&D Systems).

For Ca^2+^-imaging, the cells were pre-differentiated for 48 h in cell culture flasks, detached and plated at a density of 20,000 cells and 30,000 cells per well on 0.1% PEI-coated 384-well and 96-well plates (Greiner Bio-One), respectively, for the Ca^2+^-imaging. The cells were further differentiated for another 7 days. 50% of the medium was exchanged every 2–3 days.

### Cell culture of SH-SY5Y cells

SH-SY5Y cells were cultured as previously described (Attoff et al. 2016). Briefly, they were cultured in MEM supplemented with 10% fetal bovine serum (Gibco, 31330095), 1% non-essential amino acid solution (Gibco, 11140035), 2 mM L-glutamine (Gibco, 25030024), 100 μg/ml streptomycin, and 100 U/ml penicillin (Gibco, 15140122). For maintenance culture, SH-SY5Y cells were seeded at 27,000 cells/cm^2^ in 75 cm^2^ cell culture flasks (Corning). The cells were passaged once a week using TrypLE Express Enzyme (Gibco). SH-SY5Y cells were differentiated into a neuronal-like phenotype by exchanging the maintenance medium with differentiation medium consisting of DMEM/F12 (Gibco, 31330095) supplemented with 1 mM L-glutamine (Gibco, 25030024), 100 μg streptomycin/mL, 100 U penicillin/mL, 1 × N2-supplement (Gibco, 17502048) and 1 µM all-trans retinoic acid (RA, Sigma, R2625) 24 h after seeding. The cells were incubated in 100% humidity at 37 °C in air with 5% CO_2_.

### LUHMES Ca^2+^-imaging

Ca^2+^-imaging was performed using HT Functional Drug Screening System FDSS/µCELL (Hamamatsu Photonics) at nominal 37 °C. The FDSS/µCell system enables the indirect recording of changes of [Ca^2+^]_i_ via a Ca^2+^-sensitive fluorescent dye. The fluorescence signal of a complete 384-well plate is acquired at once with a high-speed and high-sensitivity digital ImagEM X2 EM-CCD camera (Electron Multiplying Charge-Coupled Device, Hamamatsu Photonics), but with limited spatial resolution. Therefore, the software only determines the mean fluorescence signal of each well rather than of individual cells. For compound application, the integrated dispenser head with 384 pipette tips was used, which can add the test compound to all wells simultaneously. Cells were preincubated with Cal-520 AM (AAT Bioquest) at a concentration of 1 µM for 1 h at 37 °C. For recording, the medium was exchanged by a buffer solution containing [mM]: 135 NaCl, 5 KCl, 0.2 MgCl_2_, 2.5 CaCl_2_, 10 HEPES, and 10 D-glucose, pH 7.4. Test compound application was executed after obtaining a 1.5 min baseline recording. Where applicable, a second application was executed 4.5 min after the first application. The total recording never exceeded 8 min.

For Ca^2+^-imaging experiments with a higher resolution on the single-cell level, the Cell Observer (Carl Zeiss Microscopy) was used. The Ca^2+^-sensitive dye, the cell handling before the experiment, and the buffer were the same as described above for the experiments with the high-throughput FDSS/µCELL system. The recordings were performed with 2 × 2 binning and a 42 ms exposure time. The compounds were applied after a baseline recording of at least 10 s.

### Ca^2+^ measurements in SH-SY5Y

To measure acute changes in the average [Ca^2+^]_i_ of a population, SH-SY5Y cells were examined in the 96-well plate fluorescence reader FlexStation II (Molecular Devices) using the fluorophore Fura-2AM. SH-SY5Y (35,000 cells/well; 109,375 cells/cm^2^) were seeded in maintenance culture medium in black 96-well plates with clear bottom (Corning, #3603). 24 h after seeding, maintenance medium was replaced with differentiation medium. After 72 h of differentiation, Fura-2AM dissolved in DMSO and diluted in KRH buffer (125 mM NaCl, 5 mM KCl, 1.2 mM MgSO_4_, 1.2 mM KH_2_PO_4_, 2.0 mM CaCl_2_, 6.0 mM D-glucose, and 25 mM HEPES (free acid), pH adjusted to 7.4 by 1.0 M NaOH) were added to the medium to a final concentration of 4 µM (Gustafsson et al. 2010). The plates were incubated for 30 min at 37 °C before cells were washed once with 200 µl KRH buffer. 90 µl of KRH buffer without or with 10 µM PNU-120596 (PNU) and/or 125 µM mecamylamine (Mec) and/or test chemicals in different concentrations for antagonist experiments were added to the Fura-2AM-loaded cells. The plate was again incubated for 20 min to allow full hydrolysis of the AM group before the experiment. The fluorescence was assessed at 37 °C in the fluorescence plate reader (FlexStation II; Molecular Devices) at two different excitation wavelengths, 340 nm for Ca^2+^-bound Fura-2 and 380 nm for free Fura-2, and at 510 nm emission, every 3.1 s using bottom read settings. After 26–29 s of initial baseline recording of the fluorescence intensity, 10 µl of the compound dilution (10 times higher than the final concentration to the cells) was transferred automatically by the FlexStation II (“Flex mode”) to the cell plate wells (five wells per concentration) and the fluorescence intensity was monitored for another 150 s. The ratio of fluorescence intensity at 340/380 nm was determined and the mean values from the baseline recording before the addition of test compounds was set to zero. The acute change in the Ca^2+^ influx after the addition of the compounds was quantified as the area under the curve (AUC) using the SoftMax Pro 4.8 software (Molecular Devices). All test compounds were dissolved in DMSO. Compounds were diluted in KRH buffer in 1:3 series, with 100 µM as the highest concentration. As a negative control, 0.1% DMSO in KRH buffer was used. Nicotine (11 µM) and KCl (30 mM) in KRH were used as positive controls. The Ca^2+^ influx induced by DN-IMI was normalized to the response triggered by nicotine (11 µM) or KCl (30 mM).

### Oocyte recordings

The human α3 (GenBank: U62432.1), α4 (GenBank: L35901.1, silent base exchanges to reduce GC content), β2 (GenBank: X53179.1), and β4 (GenBank: U48861.1) nAChR subunits were synthesized using FragmentGene service by Genewiz company and subsequently cloned in the pNKS2 vector (Gloor et al. 1995) using Gibson Assembly. The human α7 nAChR subunit was cloned in the pCDNA3.1 vector.

For the generation of the mRNA for injection, the plasmid DNAs of α3, α4, β2, and β4 were linearized with the NotI restriction endonuclease (New England Biolabs) and the plasmid DNA of α7 was linearized with the XbaI restriction endonuclease (New England Biolabs). The mRNAs of α3, α4, β2, and β4 were generated by in vitro transcription using the mMESSAGE mMACHINE SP6 Transcription Kit (Invitrogen). For the generation of α7 mRNA, the mMESSAGE mMACHINE T7 Transcription Kit (Invitrogen) was used. For the separation of the DNA and mRNA, a phenol–chloroform extraction (Chomczynski and Sacchi 2006) was performed. The mRNA was then obtained by ethanol precipitation from the aqueous phase; for quantification, the BioPhotometer (Eppendorf) was used.

The recordings of human α7, α3β4, α4β2, and α4β4 nAChRs expressed in *Xenopus laevis* oocytes (EcoCyte Bioscience) were performed in two-electrode voltage-clamp mode using the Roboocyte2 system and the corresponding software (version 1.4.1; Multi Channel Systems MCS). Prior to the recordings, the oocytes were maintained at 19 °C in modified Barth’s solution containing [mM]: 88 NaCl, 1 KCl, 0.33 Ca(NO_3_)_2_, 0.82 MgSO_4_, 2.4 NaHCO_3_, 0.41 CaCl_2_, 5 Tris, 100 U/ml penicillin, 100 µg/ml streptomycin, pH 7.4.

To express human α7 nAChR, we injected 50 nl of mRNA solution (30 ng mRNA) per oocyte, using the Roboinject and the corresponding software (version 1.2.1; Multi Channel Systems MCS). The subunits of the heteromeric human α3β4 nAChRs and high-sensitivity (HS) (α4)_2_(β2)_3_ combination were injected in a ratio of 1:10 (α:β subunit) with an mRNA amount of 0.33 ng of α3 and 3.33 ng of β4 for α3β4, and 3 ng of α4 and 30 ng of β2 for α4β2. The mRNA for the subunits of the low-sensitivity (LS) (α4)_3_(β2)_2_ stoichiometry was injected in a ratio of 10:1 (α:β subunit) with 10 ng of α4 and 1 ng of β2. The subunits of the α4β4 nAChR subtype were injected in a ratio of 1:1 with 3.33 ng of α4 and 3.33 ng of β4. After mRNA injection, the oocytes were maintained for 3–6 days before recordings were performed. The experiments were executed in a ND96 buffer solution containing [mM]: 96 NaCl, 2 KCl, 1 MgCl_2_, 1.8 CaCl_2_, 5 HEPES, pH 7.4. The oocyte membrane potential was kept at − 50 mV in all recordings. In experiments with α7, the compounds were applied for 5 s followed by a 60 s wash period. At the end of each recording, a reference application of 1 mM nicotine was performed. In experiments with heteromeric nAChRs, the compound was applied for 3 s, followed by a washout of 10 s, and an application of acetylcholine (ACh) for 1 s, which was followed by a washout of 60 s. The recordings for α3β4, α4β2 (HS), α4β2 (LS) and α4β4 were performed with 200 µM, 3 µM, 100 µM and 100 µM ACh, respectively. ACh was applied as an additional reference for run-down detection and positive control. Therefore, ACh was applied four times before the addition of the first compound concentration and after the application of each compound concentration. After the measurement of all compound concentrations, the last application was a reference exposure to nicotine with 1 mM for α3β4, 10 µM for α4β2 (HS), 100 µM for α4β2 (LS) and 100 µM for α4β4. The reference response triggered by nicotine was used for the normalization of the compound effects.

For the antagonist experiments, DN-IMI was applied for 3 s after a 5 s baseline period. The application of DN-IMI was followed by a wash period of 70 s. At first, four control recordings were performed, followed by three recordings in the presence of each of the three antagonist concentrations in ascending order. Finally, three recordings were executed during the washout. DN-IMI was applied at 1 µM in recordings with α4β2 (HS) and at 30 µM in recordings with α3β4 and α7.

### Physiologically based toxicokinetic modeling

A physiology-based toxicokinetic (PBTK) model for DN-IMI was established in the Simcyp Simulator V20 (Certara) using a previously published approach (Albrecht et al. 2019). Due to the lack of published human metabolism and exposure data for DN-IMI, an analog approach was used to inform the PBTK model using a compound, in our case atenolol, with known human pharmacokinetics and similar physicochemical properties. The input parameters for DN-IMI and atenolol are given in Table S8 and further details are found in Fig. S10.

### Data analysis

For the high-throughput Ca^2+^-imaging data obtained in LUHMES cells, an offset correction using the FDSS software (version 3.2) was performed. Afterward, the data were exported and further analyzed with scripts written in R (version 3.6.3) (R Core Team 2020). The concentration–response curves were fitted using a log-logistic model described by Ritz et al. (2015), utilizing the R package *drc* with its function *drm()* and *LL2.2()* with the following equation: *f*(*x*) = *d*/[1 + exp(*b*(log(*x*) − *ẽ*))] (Ritz et al. 2015). The logarithm of the half-maximal effective concentration (logEC_50_) between 0 and the upper limit (*d*), which was set to 1, is represented by *ẽ*, *x* denotes the concentration, and b stands for the slope parameter (Ritz et al. 2015). In cases with normalizations to responses induced by other compounds, the function *LL2.3()* was used with a variable upper limit (*d*; Ritz et al. 2015). The same equation was used to determine the half-maximal inhibitory concentration (logIC_50_). Then the logEC_50_ and logIC_50_ values were converted into the pIC_50_ and pEC_50_ values, which are the negative logarithms to base 10.

Concentration–effect responses in the SH-SY5Y [Ca^2+^]_i_, were analyzed by the GraphPad Prism8.0 software using the four-parameter sigmoidal curve fit settings and the concentrations giving 50% increase in [Ca^2+^]_i_ in relation to the nicotine response were estimated.

The single-cell Ca^2+^-imaging recordings were exported and analyzed in Fiji ImageJ (version 1.52i) to get the average fluorescence signal of each cell. These signals were further analyzed in R, where a threshold detection was performed to detect responding cells. For this, the offset was corrected by subtracting the mean of 20–65% of the fluorescence signal of the pre-application period from the recording, to be robust against spontaneous activity. The threshold was defined as mean + 3 * SD of the negative control recordings, during the detection phase of 6.5 s during the application.

The baseline correction of voltage-clamp oocyte recordings was performed with the Roboocyte2 + software (version 1.4.3; Multi Channel Systems MCS, Germany). The maximal current influx and further analysis were executed in scripts written in R. In the antagonist experiments with oocytes, the maximal inward current was determined for the last response of each period (control, three antagonist concentrations, and washout).

The following R packages were utilized for data handling: cowplot (Wilke 2019), dplyr (Wickham et al. 2020), drc (Ritz et al. 2015), ephys2 (Danker 2018), ggplot2 (Wickham 2016), htmlwidgets (Vaidyanathan et al. 2019), lemon (Edwards 2019), magick (Ooms 2020), magrittr (Bache and Wickham 2014), matrixStats (Bengtsson 2020), modelr (Wickham 2020), multcomp (Hothorn et al. 2008), plotrix (Lemon 2006), proto (Grothendieck et al. 2016), and tidyverse (Wickham et al. 2019).

Unless mentioned differently, values are presented as means ± SEM. Experiments were usually performed with at least three technical replicates per condition. Detailed data on pEC_50_, pIC_50_, and numbers of experimental repetitions are given in supplementary tables. Unless mentioned differently, statistical significance was defined as *P* < 0.05 and was determined by one-way ANOVA with Dunnett’s post hoc test as indicated. To determine benchmark concentrations (BMC), and their upper and lower 95% confidence intervals (BMCL, BMCU), the BMC online software of UKN was used (Krebs et al. 2020).

## Results and discussion

### Activation, inhibition, and desensitization of nAChRs functionally expressed in LUHMES and SH-SY5Y cells

#### Activation of nAChRs on LUHMES cells by DN-IMI

**Fig. 1:**
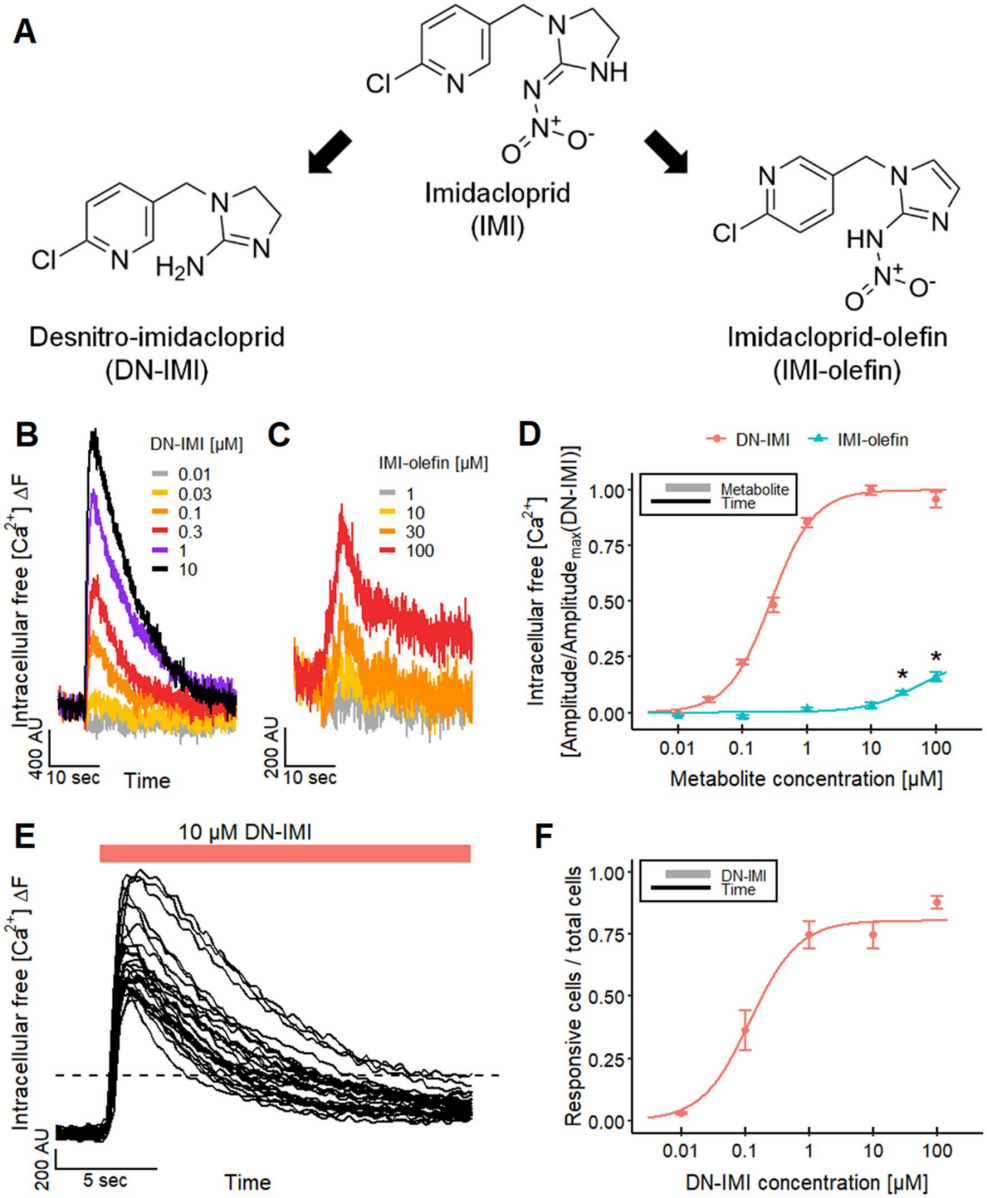
Effect of desnitro-imidacloprid (DN-IMI) and imidacloprid-olefin (IMI-olefin) on LUHMES neurons. **A** Chemical structures of imidacloprid (IMI) and its two metabolites desnitro-imidacloprid (DN-IMI) and imidacloprid-olefin (IMI-olefin). **B**–**F** LUHMES neurons were differentiated for 9 days before they were loaded with an [Ca^2+^]_i_ indicator dye and used for Ca^2+^-imaging. **B**, **C** The cells cultivated in 384-well plates were exposed to various concentrations of DN-IMI and IMI-olefin, and exemplary recordings of the fluorescence signal from a whole well are shown. **D** The fluorescence data (peak amplitude) of multiple experiments were quantified and normalized to the maximal response stimulated by DN-IMI (means ± SEM are displayed). The significance of the responses triggered by IMI-olefin was determined between control recordings and the responses evoked by IMI-olefin (*: *p* < 0.05). For DN-IMI, a sigmoid curve was fitted to the data, and a pEC_50_ value of 6.6 ± 0.03 was obtained as a potency estimate. Note the treatment scheme (upper left corner), illustrating the experimental design. **E**, **F** LUHMES cultures in 96-well plates were used to image the [Ca^2+^]_i_ responses of single cells with a fluorescent microscope. Regions of interest were assigned for all individual cell bodies in the image section. **E** Ca^2+^-imaging traces of the responses of individual cell bodies are shown after exposure to DN-IMI (10 µM). **F** The percentage of cells that responded with a clear fluorescence increase (= rise in [Ca^2+^]_i_) to different concentrations of DN-IMI was determined. Note the treatment scheme (upper left corner), illustrating the experimental design. Detailed data on n numbers are listed in Tables S6

LUHMES neurons express functional α7 and non-α7 nAChRs, and they have proven useful for the characterization of different neonicotinoids like IMI by high-throughput Ca^2+^-imaging (Loser et al. 2021a). We used this system here for the functional characterization of the two IMI metabolites DN-IMI and IMI-olefin (Fig. 1A). Both metabolites produced clear signals (Fig. 1B, C). A quantification of [Ca^2+^]_i_ responses yielded a pEC_50_ of 6.6 for DN-IMI (Fig. 1D). DN-IMI appeared at least as potent as ACh and nicotine (pEC_50_ values of ~ 6.0 in LUHMES neurons) (Loser et al. 2021a). These signaling data are in line with published binding data that suggest a similar affinity of DN-IMI and nicotine for mammalian nAChRs (Tomizawa and Casida 1999; D’Amour and Casida 1999; Tomizawa et al. 2000).

For IMI-olefin, the pEC_50_ was not reached within the measurement range (≤ 100 µM), but a significant response (around 15% of the maximal response to DN-IMI) was found at 30–100 µM. Thus, the potency of IMI-olefin was similar to that of its parent compound IMI (Loser et al. 2021a). Our observations on signaling potency are consistent with the literature data for differences in binding affinity to mammalian nAChRs (Chao and Casida 1997; Tomizawa and Casida 1999; D’Amour and Casida 1999; Tomizawa et al. 2000).

The data on DN-IMI were confirmed by a different analytical method. Instead of the whole-culture-based high-throughput [Ca^2+^]_i_ assay, we used traditional time-lapse fluorescence microscopy to quantify responses of individual cells (Fig. 1E). We found here a percentage of responsive cells of ~ 80%. This population was similar in size to that measured in a previous study, using nicotine as a stimulus (Loser et al. 2021a). The quantification of single-cell responses confirmed the sub-micromolar potency of DN-IMI and suggested that the majority of all cells responded functionally to the IMI metabolite (Fig. 1F).

#### Activation of α7 and non-α7 nAChRs on LUHMES and SH-SY5Y cells by DN-IMI

**Fig. 2:**
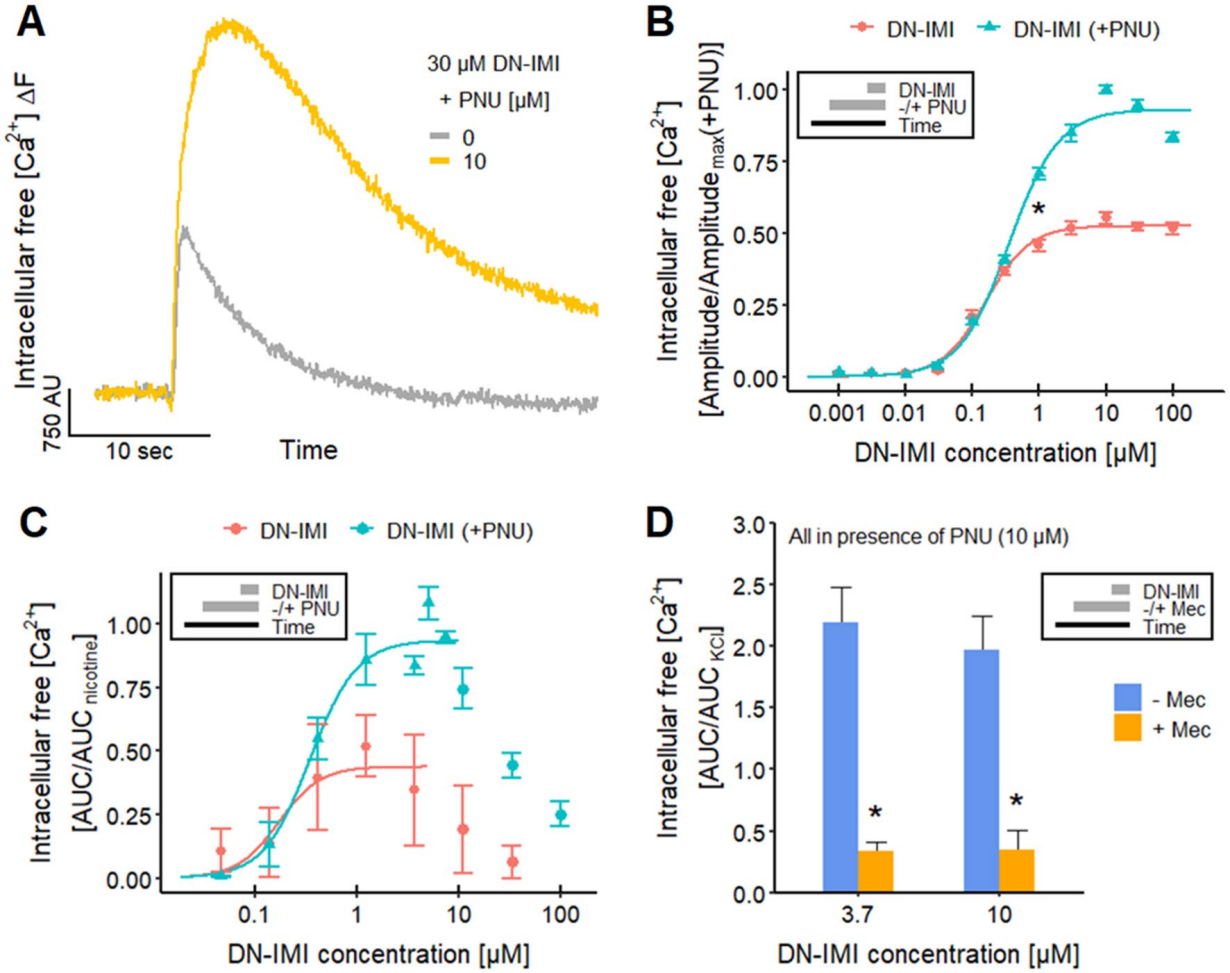
Activation of human α7 nAChRs on LUHMES and SH-SY5Y cells by DN-IMI. **A**, **B** LUHMES neurons differentiated in 384-well plates were exposed to various concentrations of DN-IMI in the absence and presence of PNU-120596 (PNU, 10 µM), a selective positive allosteric modulator of α7 nAChRs. **A** Representative recordings of the Ca^2+^-imaging fluorescence signal from a whole well are shown. **B** The fluorescence data (peak amplitude) of multiple experiments were quantified and normalized to the maximal response triggered by DN-IMI in the presence of PNU (means ± SEM are displayed). After sigmoidal curve fitting, the relative half maximum (turning point) was determined: they were on a -log(M) scale: 6.8 ± 0.04 in the absence of PNU and 6.5 ± 0.03 in the presence of PNU. The upper asymptote was at 53% of the maximal response (found in all experiments at all conditions) in the absence of PNU and at 93% in the presence of PNU. The significance of the difference between the effects of DN-IMI (1 µM) in the absence and presence of PNU was evaluated (*: *p* < 0.05). Detailed data on n numbers are found in Table S6. **C**, **D** SH-SY5Y cells were used for automated [Ca^2+^]_i_ monitoring, with the area under the curve (AUC) of the fluorescence intensity as assay endpoint. Data were normalized to a reference signal (10 µM nicotine in **C**, 30 mM KCl in **D**). All data are from multiple experiments and are displayed as means ± SEM. **C** Data were obtained for multiple concentrations of DN-IMI in the absence and presence of PNU, and the ascending arms of the curves were fitted for concentrations < 10 µM. The sigmoidal curve fit yielded relative pEC_50_s of 6.8 ± 0.36 in the absence of PNU (estimated maximum at ~ 0.44, *n* = 5) and 6.5 ± 0.07 (0.3 µM, estimated maximum at ~ 0.93, *n* = 4) in the presence of PNU. **D** The [Ca^2+^]_i_ response of SH-SY5Y cells triggered by DN-IMI [in the presence of PNU (10 µM)] was measured in the absence and presence of the nAChR antagonist mecamylamine (Mec, 125 µM) (*n* = 5); *: *p* < 0.05. Note the treatment schemes, illustrating the experimental design

There is a large variety of nAChR subtypes with distinct functions in the nervous system. To get initial information, we examined whether the human α7 nAChR is affected by DN-IMI. This Ca^2+^ permeable receptor is widely distributed in the central nervous system and involved in the modulation of neurotransmitter release (McGehee et al. 1995; Gray et al. 1996; Alkondon et al. 1999; Gotti et al. 2006; Zoli et al. 2015). We utilized PNU-120596 (PNU), a selective positive allosteric modulator of the α7 nAChR, to slow down the α7 nAChR inactivation and enable thereby the detection of the α7 nAChR-mediated response in Ca^2+^-imaging (Hurst et al. 2005; Dickinson et al. 2007; Ng et al. 2007; Grønlien et al. 2007; Papke et al. 2009; Williams et al. 2011; Chatzidaki et al. 2015; Larsen et al. 2019). The response of LUHMES neurons to DN-IMI was strongly enhanced and prolonged in the presence of PNU (Fig. 2A). A quantification at multiple DN-IMI concentrations showed that this effect is less pronounced at sub-maximal receptor stimulation (Fig. 2B). The maximal amplitude triggered by DN-IMI was increased by PNU by around 40%. This strongly suggests the activation of α7 nAChRs. These data are fully in line with findings showing the enhancement of neonicotinoid effects by PNU in LUHMES neurons (Loser et al. 2021a). The activation of non-α7 nAChRs at low concentrations of DN-IMI (0.03–0.3 µM) is most likely the reason for an absence of PNU enhancement in the low concentration range.

To further support these findings, we examined the effect of DN-IMI on a second human cell system. SH-SY5Y neuroblastoma cells predominantly express the α7 nAChR subtype, together with α3-containing receptors (Loser et al. 2021a). Therefore, they show little response to neonicotinoids or nicotine in the absence of PNU, and also DN-IMI only triggered small responses reaching about 44% of the maximal response obtained in the presence of PNU (Fig. 2C). In this experimental setup (presence of PNU), DN-IMI led to a strong, concentration-dependent [Ca^2+^]_i_ response with half-maximal responses at about 0.3 µM, a peak at ~ 3 µM, and declining responses at even higher concentrations (Fig. 2C). The maximal response triggered by DN-IMI was roughly similar to the one evoked by nicotine. The strong signal increase in the presence of PNU is in line with our results for LUHMES.

We also examined the effect of IMI-olefin in the absence and presence of PNU on [Ca^2+^]_i_ of LUHMES neurons (Fig. S1A), and we observed a strong enhancement of the signal. This allowed for the determination of a pEC_50_ (5.5 in the presence of PNU) (Fig. S1B). The significant increase of the responses indicates the activation of human α7 nAChRs by IMI-olefin, but with a significantly lower potency compared to DN-IMI.

#### Inhibition of DN-IMI-evoked responses of LUHMES and SH-SY5Y cells by nAChR antagonists

**Fig. 3:**
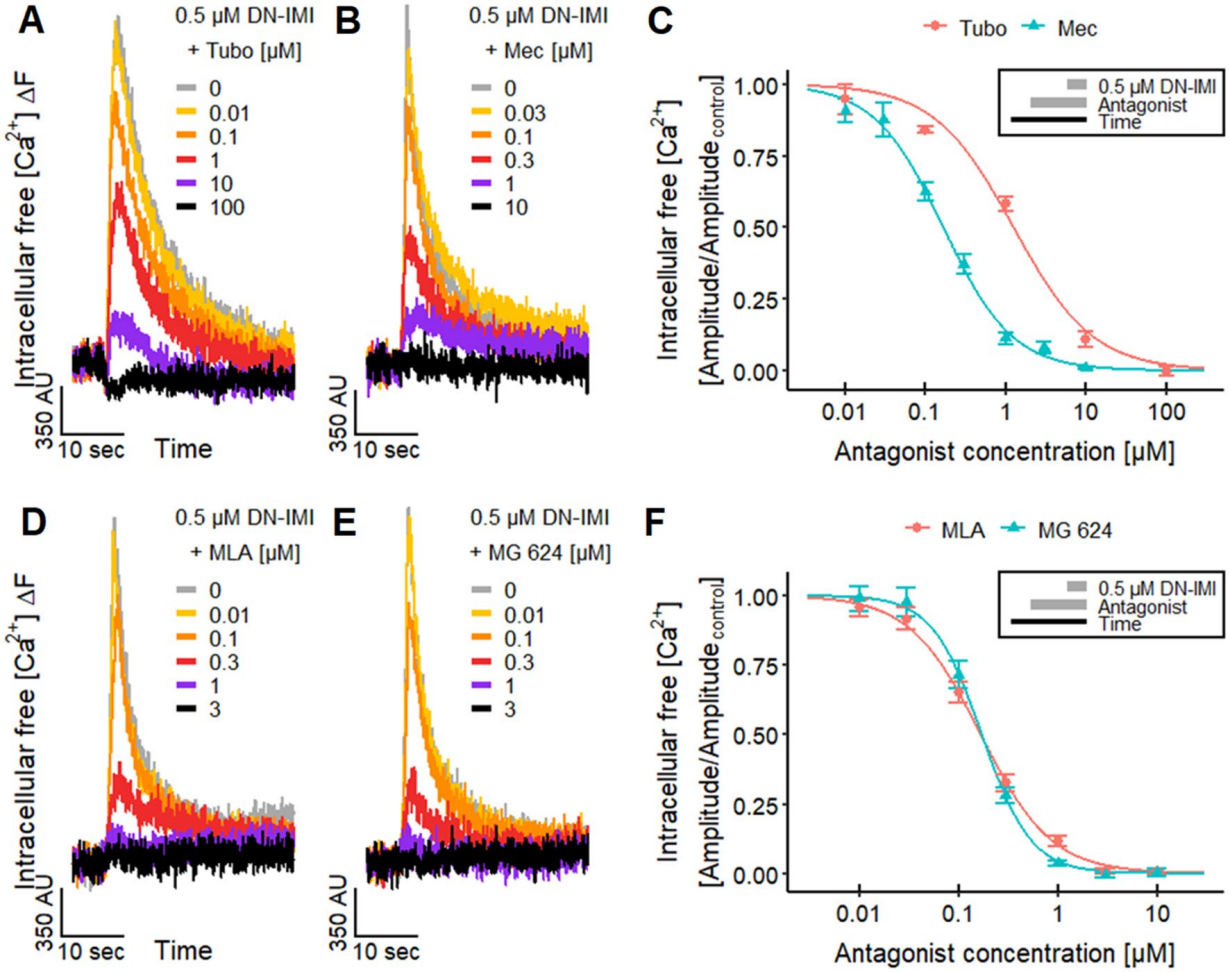
Inhibition of DN-IMI signaling by nAChR antagonists. LUHMES neurons differentiated in 384-well plates were pretreated with various concentrations of nAChR antagonists before DN-IMI (0.5 µM) was applied in Ca^2+^-imaging experiments. **A**, **B** Exemplary recordings of the fluorescence signal from a whole well are shown for the effects of **A** tubocurarine (Tubo) and **B** mecamylamine (Mec) on the responses evoked by DN-IMI. **C** The fluorescence data (peak amplitude) of multiple experiments were quantified and normalized to control recordings (means ± SEM are displayed). After curve fitting, pIC_50_ values of 5.9 ± 0.05 for Tubo and 6.8 ± 0.03 for Mec were determined for their inhibitory effects on DN-IMI-induced [Ca^2+^]_i_ responses. Note the treatment scheme (upper right corner), illustrating the experimental design. **D**, **E** Exemplary recordings of the fluorescence signal from a whole well are shown for the effects of **D** MLA and **E** MG 624 on the [Ca^2+^]_i_ responses evoked by DN-IMI. **F** The fluorescence data (peak amplitude) of multiple experiments were quantified and normalized to control recordings (means ± SEM are displayed). After curve fitting, pIC_50_s of 6.8 ± 0.03 for MLA and 6.8 ± 0.03 for MG 624 were determined. Note the treatment scheme (upper right corner), illustrating the experimental design. Detailed data on n numbers are found in Table S6

We used a pharmacological approach to verify that the signaling ([Ca^2+^]_i_) effect of DN-IMI is mediated exclusively by nAChRs. For this purpose, LUHMES cells were pretreated with several well-known nAChR antagonists. Tubocurarine (Tubo) (Jonsson et al. 2006) antagonized the responses evoked by DN-IMI with a pIC_50_ of 5.9 (Fig. 3A, C). Tubo completely blocked the response at 100 µM, indicating that the entire DN-IMI-evoked Ca^2+^-signaling was mediated by nAChRs. The obtained pIC_50_ value is comparable to the values determined for several nAChR agonists in experiments with LUHMES (Loser et al. 2021a).

To further substantiate this finding, we utilized the non-competitive nAChR antagonist mecamylamine (Mec) (Papke et al. 2008; Capelli et al. 2011). It blocked the DN-IMI-induced response in LUHMES neurons with a pIC_50_ of 6.8 (Fig. 3B, C), which is in line with the literature data of 6.6 for human α3β2 nAChRs (Chavez-Noriega et al. 2000). We also used Mec in the SH-SY5Y cultures, and the [Ca^2+^]_i_ responses induced by DN-IMI were strongly blocked (Fig. 2D). This confirmed that also in this cell model, DN-IMI signaling was strictly dependent on nAChRs.

To further investigate the agonism of DN-IMI on nAChRs on LUHMES, we researched the effect of the antagonist methyllycaconitine (MLA), which is highly potent (low nM range) on α7 nAChRs compared to other nAChR subtypes (Puchacz et al. 1994; Gopalakrishnan et al. 1995; Palma et al. 1996; Buisson et al. 1996; Capelli et al. 2011). MLA inhibited the response to DN-IMI with a pIC_50_ of 6.8 (Fig. 3D, F), which is comparable to the value obtained for nicotine with LUHMES (Loser et al. 2021a). The pIC_50_ is similar to the literature data for human α4β2 and α6-containing (α6/3β2β3) nAChRs (Capelli et al. 2011). This (relatively low) potency of MLA in LUHMES indicates the involvement of non-α7 nAChRs in the [Ca^2+^]_i_ response evoked by DN-IMI.

Finally, we applied the nAChR antagonist MG 624 (Gotti et al. 2000; Capelli et al. 2011) on LUHMES neurons. The resulting pIC_50_ of 6.8 (Fig. 3E, F) is comparable to the pIC_50_ of nicotine obtained with LUHMES neurons (Loser et al. 2021a) and previously reported data for α4β2, α3β4, α7, and α1β1δε nAChRs (Capelli et al. 2011).

In summary, the antagonist data demonstrate the activation of nAChRs by DN-IMI and indicate the involvement of different nAChR subtypes.

#### Desensitization of cholinergic responses of LUHMES and SH-SY5Y cells by DN-IMI

**Fig. 4:**
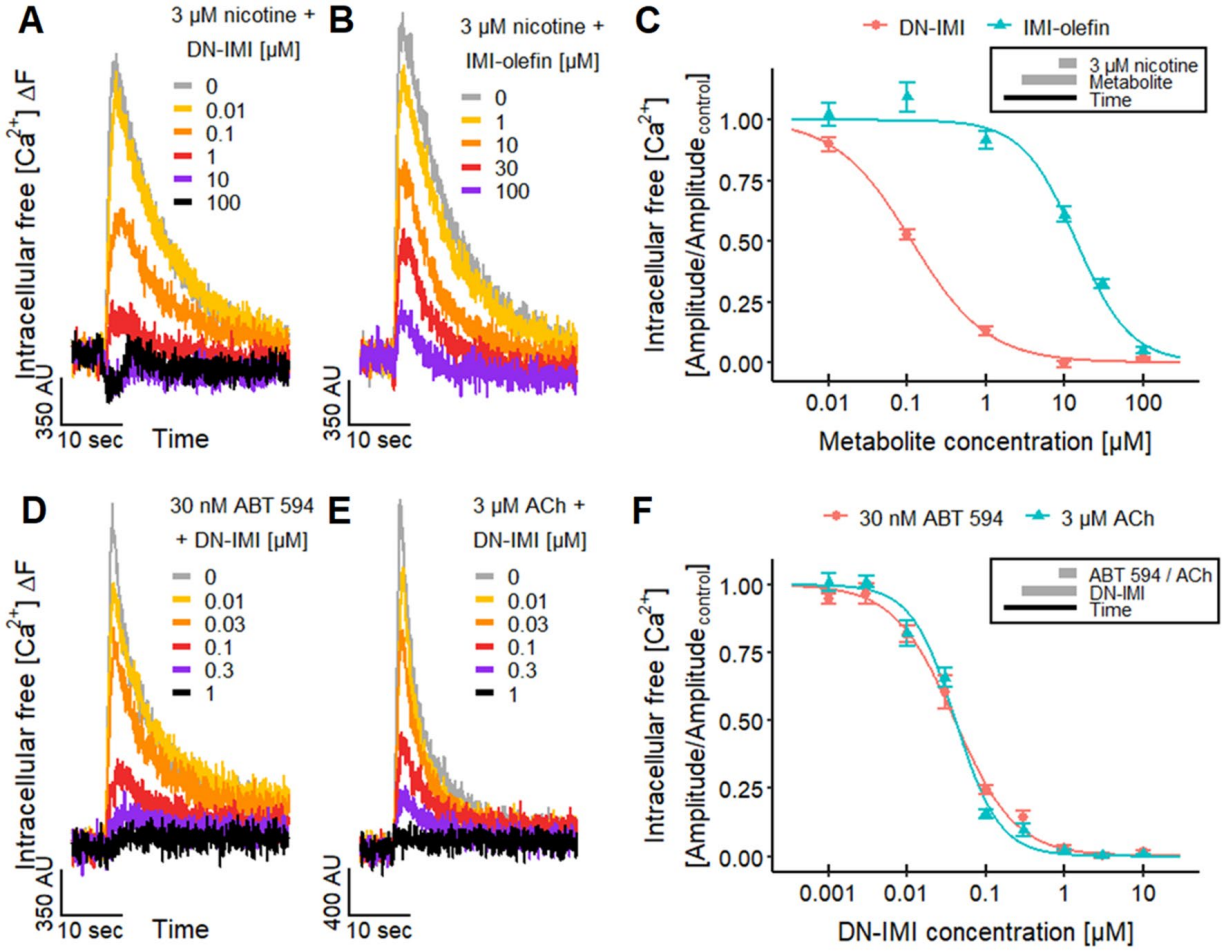
Desensitizing effects of DN-IMI and IMI-olefin on nAChRs. LUHMES neurons differentiated in 384-well plates were pretreated with various concentrations of DN-IMI or IMI-olefin for 4.5 min before different nAChR agonists were applied and [Ca^2+^]_i_ signals were recorded. **A**, **B** Exemplary traces of the fluorescence signal from a whole well are shown for the desensitizing effects of different concentrations of **A** DN-IMI and **B** IMI-olefin on the [Ca^2+^]_i_ responses evoked by nicotine. **C** The fluorescence data (peak amplitude of 3 µM nicotine) of multiple experiments were quantified and normalized to control recordings (means ± SEM are displayed). After curve fitting, pIC_50_ values of 6.9 ± 0.03 for DN-IMI and 4.9 ± 0.03 for IMI-olefin were determined. Note the treatment scheme (upper right corner), illustrating the experimental design. **D**, **E** Exemplary traces of the fluorescence signal are shown for the effects of DN-IMI on the responses evoked by **D** 30 nM ABT 594 and **E** 3 µM ACh. **F** The fluorescence data (peak amplitude of the agonist stimulus) of multiple experiments were quantified and normalized to control recordings (means ± SEM are displayed). After curve fitting, pIC_50_ values of 7.4 ± 0.03 (ABT 594) and 7.4 ± 0.03 (ACh) were determined. Note the treatment scheme (upper right corner), illustrating the experimental design. Detailed data on n numbers are found in Table S6

An important feature of nAChRs is desensitization. This is the inactivation of the receptor during agonist exposure or upon closely timed repeated agonist applications. Thus, even in the presence of an agonist, the receptor can stop the signaling and may not be activated again within a certain period after an initial stimulation (Fenster et al. 1997; Quick and Lester 2002; Paradiso and Steinbach 2003; Lester 2004; Rollema et al. 2010; Marks et al. 2010; Capelli et al. 2011; Papke et al. 2011; Campling et al. 2013; Eaton et al. 2014; Arias et al. 2015; Rollema and Hurst 2018). The desensitization of a receptor is typically caused by an agonist concentration that activates the receptor, but it can also occur at low concentrations that are not sufficient to activate it (Fenster et al. 1997; Paradiso and Steinbach 2003; Lester 2004; Rollema et al. 2010; Capelli et al. 2011; Arias et al. 2015; Rollema and Hurst 2018). As our previous results indicate an agonistic effect of both IMI metabolites, we investigated whether they would also desensitize the nAChRs on LUHMES neurons. In these experiments, the metabolites were pre-applied at various concentrations and then the response of LUHMES neurons was triggered by the exposure to nicotine and measured by Ca^2+^-imaging. The pretreatment led to a pronounced reduction of the nicotinic signaling (Fig. 4A, B). The corresponding concentration–response curves yielded pIC_50_ values of 6.9 for DN-IMI and 4.9 for IMI-olefin (Fig. 4C). The pIC_50_ of IMI-olefin is comparable to the effects of its parent compound IMI and other neonicotinoids (Loser et al. 2021a). The pIC_50_ of DN-IMI is comparable to pIC_50_ values reported for the desensitizing effect of nicotine on human α4β2, α4β4, and α3β4 nAChRs (Fenster et al. 1997; Lester 2004; Capelli et al. 2011). Thus, DN-IMI was more potent than several neonicotinoids (pIC_50_s of ~ 5.4) (Loser et al. 2021a) and IMI-olefin at attenuating the response evoked by nicotine. For confirmation of the desensitization in a different cell model, we used SH-SY5Y cells. In addition, here, pretreatment with DN-IMI reduced/abolished the response to nicotine in the submicromolar range. This effect was clearly more potent than the desensitization observed by IMI and another neonicotinoid pesticide, acetamiprid (Fig. S2). Thus, desensitization by neonicotinoids was confirmed in a second cell model, and the particularly high potency of DN-IMI was reproduced.

To confirm that the desensitizing effect was not specific for nicotine stimulation, we used the endogenous nAChR agonist ACh and the selective non-α7 nAChR agonist ABT 594 for stimulation (Donnelly-Roberts et al. 1998; Michelmore et al. 2002) (Fig. 4D, E). Here, we observed pIC_50_ values of ~ 7.4 for the desensitization (Fig. 4F). This high potency is in agreement with other observations that desensitization of nAChR can occur at lower concentrations than required for activation (Fenster et al. 1997; Paradiso and Steinbach 2003; Lester 2004; Rollema et al. 2010; Capelli et al. 2011; Arias et al. 2015; Rollema and Hurst 2018). The large difference in potency of DN-IMI and its parent compound IMI and other neonicotinoids is consistent with the literature data for potency differences in binding assays with mammalian nAChRs (Chao and Casida 1997; Tomizawa and Casida 1999; D’Amour and Casida 1999; Tomizawa et al. 2000).

In summary, DN-IMI desensitized nAChRs in the nM range, and this may be of toxicological significance, as nAChR signaling plays an important role in the central nervous system (Alkondon et al. 1999; Champtiaux et al. 2003; Levin et al. 2006; Gotti et al. 2006; Zoli et al. 2015).

### Activation of human α4β2, α7, and α3β4 nAChRs by DN-IMI

**Fig. 5:**
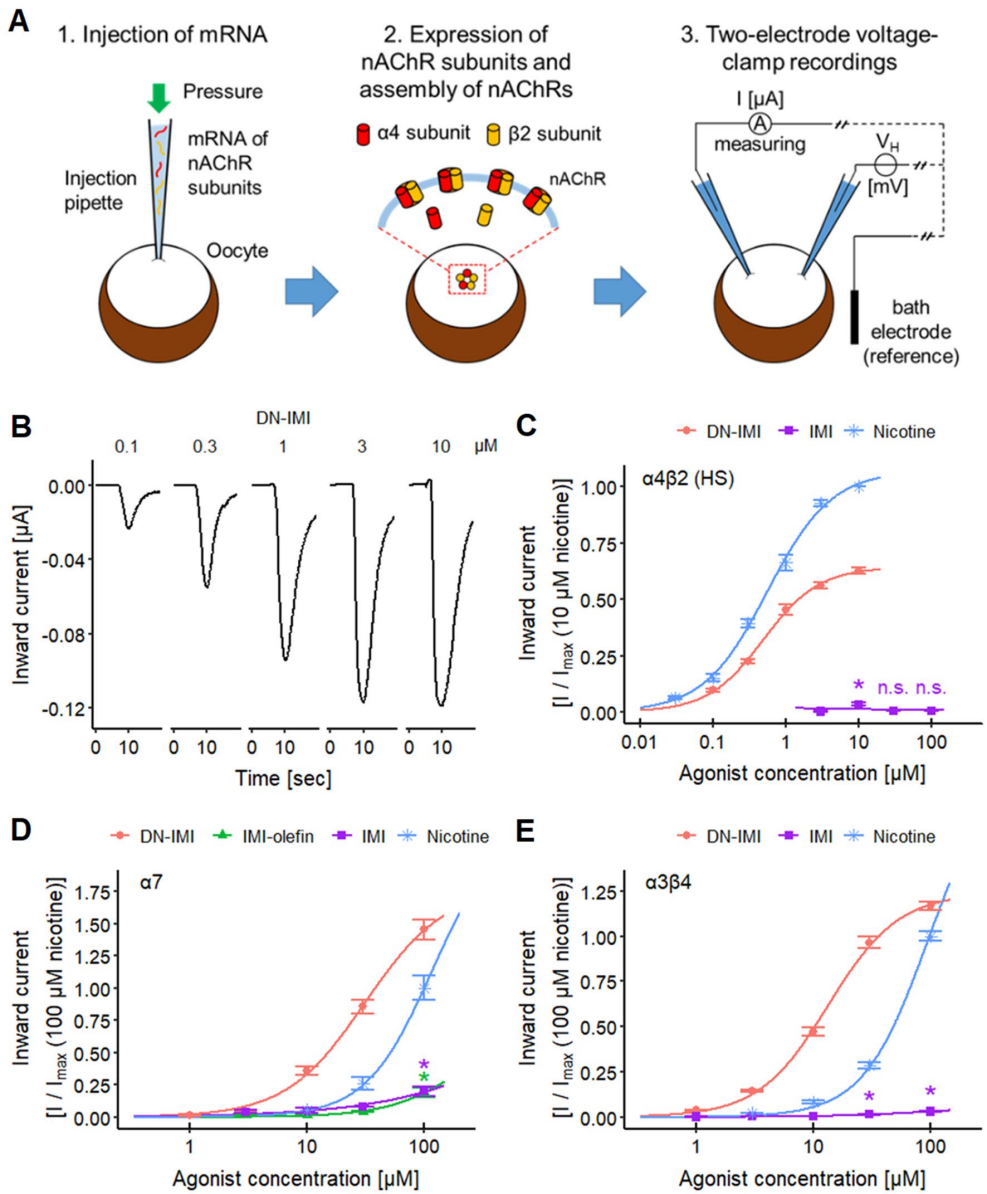
Effects of DN-IMI on human nAChR subtypes heterologously expressed by *Xenopus laevis* oocytes. **A** The basic principle of the experiments with human nAChRs heterologously expressed by *Xenopus laevis* oocytes is presented. (1) The genetic information (mRNA) of the respective nAChR subunits, in this example α4 (red) and β2 (orange), is injected at the desired ratio [here: 1 (α4):10 (β2)] into the oocytes. (2) The oocytes are incubated for a few days to allow protein expression and membrane integration as functional nAChRs. (3) The experiments were performed in two-electrode voltage-clamp recording mode. The agonist-evoked inward current through the nAChRs was measured by the current electrode, while the membrane potential of the oocyte was kept constant (*V*_H_ = − 50 mV) by a regulated voltage electrode and its reference electrode in the bath solution. **B** Increasing concentrations of DN-IMI were added to the bath solution, with washout phases between the recordings. Exemplary inward currents through human α4β2 (HS) nAChRs are shown. Note that an excess of β2 subunits was used here to generate pentameric receptors with two α subunits (designated here as high-sensitivity (HS) variant, compared to receptors with > 2 α subunits). **C** The inward current data (amplitude) of human α4β2 (HS) nAChRs heterologously expressed by *Xenopus laevis* oocytes of multiple experiments were quantified (means ± SEM are displayed). After curve fitting, relative pEC_50_ values (curve inflection point) of 6.3 ± 0.04 (estimated maximum amplitude at 64% of nicotine’s) for DN-IMI and 6.3 ± 0.04 for nicotine were determined. The significance of the responses triggered by IMI was evaluated between the lowest concentration (3 µM) and the other concentrations (*: *p* < 0.05; n.s., not significant). The inward current amplitudes were normalized to the response induced by nicotine (10 µM). Exemplary current traces of DN-IMI and nicotine are shown in Fig. S3C and S3D, respectively. **D** The inward current data (amplitude) of human α7 nAChRs heterologously expressed by *Xenopus laevis* oocytes of multiple experiments were quantified (means ± SEM are displayed), and after curve fitting relative pEC_50_ values of 4.5 ± 0.09 (estimated maximum of 83%) for DN-IMI and 3.9 ± 0.04 for nicotine were obtained. The significance of the responses triggered by IMI-olefin and IMI was evaluated between the lowest concentration (3 µM) and the other concentrations (*: *p* < 0.05). The current amplitudes were normalized to the response induced by nicotine (100 µM). Exemplary current traces of DN-IMI, IMI-olefin, and nicotine and the complete concentration–response curve for nicotine are shown in Fig. S5. **E** The inward current data (amplitude) of human α3β4 nAChRs heterologously expressed by *Xenopus laevis* oocytes of multiple experiments were quantified (means ± SEM are displayed), and after curve fitting relative pEC_50_ values of 4.9 ± 0.03 for DN-IMI and 4.0 ± 0.01 for nicotine were determined. The significance of the responses triggered by IMI was determined between the lowest concentration (1 µM) and the other concentrations (*: *p* < 0.05). The current amplitudes were normalized to the response induced by nicotine (100 µM). Exemplary current traces of DN-IMI, IMI, and the complete concentration–response curve for nicotine are shown in Fig. S6C–F. Detailed data on n numbers are found in Table S6

To verify an agonistic effect of DN-IMI on the physiologically important neuronal nAChR subtypes α4β2, α7, and α3β4, we expressed each of them in *Xenopus laevis* oocytes and performed two-electrode voltage-clamp recordings (Fig. 5A).

First, we focused our experiments on the α4β2 receptor, which can assemble in two different stoichiometries. The high-sensitivity (HS) variant (two α4 subunits and three β2 subunits) has been reported to have a pEC_50_(ACh) of ~ 5.7 in the *Xenopus laevis* oocyte expression system, while the low-sensitivity variant (three α4 subunits and two β2 subunits) had a pEC_50_(ACh) of ~ 4.1 (Bermudez and Moroni 2006; Moroni et al. 2006; Jonsson et al. 2006; Carbone et al. 2009; Mineur et al. 2009; Mazzaferro et al. 2011; Harpsøe et al. 2011; Li et al. 2011; Benallegue et al. 2013). In our system, we found for the α4β2 (HS) receptor a pEC_50_(ACh) of ~ 5.7 (Fig. S3A, B). For nicotine, we found a pEC_50_ of 6.3, in line with the literature data (Moroni et al. 2006). DN-IMI yielded a relative pEC_50_ of 6.3 (Fig. 5B, C). The data show a high potency for this nAChR subtype; our data suggest that DN-IMI has a similar potency but slightly lower efficacy (64% of full stimulation) than nicotine (Fig. S3C, D). Its parent compound IMI did not trigger a concentration-dependent activation of the receptor in the tested concentration range (≤ 100 µM). For control purposes, we applied DN-IMI (30 µM) to *Xenopus laevis* oocytes without additional receptor expression (injection of water without mRNA). In this experimental setup, we did not detect any current responses (*n* = 5, data not shown). These findings show that DN-IMI only triggered inward currents via the activation of the heterologously expressed human nAChRs. This was further confirmed by antagonist experiments, where the response of the human α4β2 (HS) receptor to DN-IMI was concentration-dependently and reversibly blocked by the non-competitive nAChR antagonist Mec (Fig. S4).

To verify an agonistic effect of DN-IMI and IMI-olefin on human α7 nAChRs, we expressed this nAChR subtype in *Xenopus laevis* oocytes and performed two-electrode voltage-clamp recordings (Figs. 5D and S5). DN-IMI had a relative pEC_50_ of 4.5 with a lower efficacy than nicotine (Figs. 5D and S5A, D). Compared to nicotine, DN-IMI thus showed a slightly higher potency and a partial agonistic effect (estimated maximum at ~ 81% of the maximal response to nicotine) on human α7 nAChRs, well in line with our Ca^2+^-imaging data (Fig. 2B, C). IMI-olefin and its parent compound IMI also stimulated significant inward currents but with a lower potency and efficacy than DN-IMI (Figs. 5D and S5B, D). The results for IMI match our previous findings with LUHMES and SH-SY5Y neurons (Loser et al. 2021a). The application of nicotine yielded a pEC_50_ of 3.9 (Figs. 5D and S5C, D), which is comparable to the literature data (Briggs et al. 1995). As an internal consistency check, we performed antagonist experiments, where the DN-IMI-triggered response of the human α7 nAChR was concentration-dependently and reversibly blocked by the selective α7 receptor antagonist MLA (Fig. S4).

As a third approach, we investigated the effects of DN-IMI on human α3β4 nAChRs expressed by *Xenopus laevis* oocytes. The application of nicotine and ACh resulted in pEC_50_s of 4.0 and 3.8 (Figs. 5E and S6A, B, D), respectively, which are both comparable to the literature data (Wang et al. 1996; Nelson et al. 2001; Jonsson et al. 2006). The addition of DN-IMI to oocytes expressing human α3β4 nAChR yielded a relative pEC_50_ of 4.9 (Figs. 5E and S6C, D). IMI evoked small but significant inward currents in a concentration-dependent manner (Figs. 5E and S6D–F). Responses of the human α3β4 nAChR triggered by DN-IMI were concentration-dependently and reversibly blocked by the nAChR antagonist Tubo (Fig. S4).

For the further characterization of DN-IMI on individual receptors, we investigated the effects of DN-IMI and IMI on the low-sensitivity variant of α4β2 (LS) and on α4β4 nAChRs. DN-IMI yielded pEC_50_s of 5.3 for α4β2 (LS) and 5.5 for α4β4 (Fig. S7). IMI did not trigger a concentration-dependent activation of these two nAChR subtypes in the tested concentration range (≤ 100 µM).

**Fig. 6:**
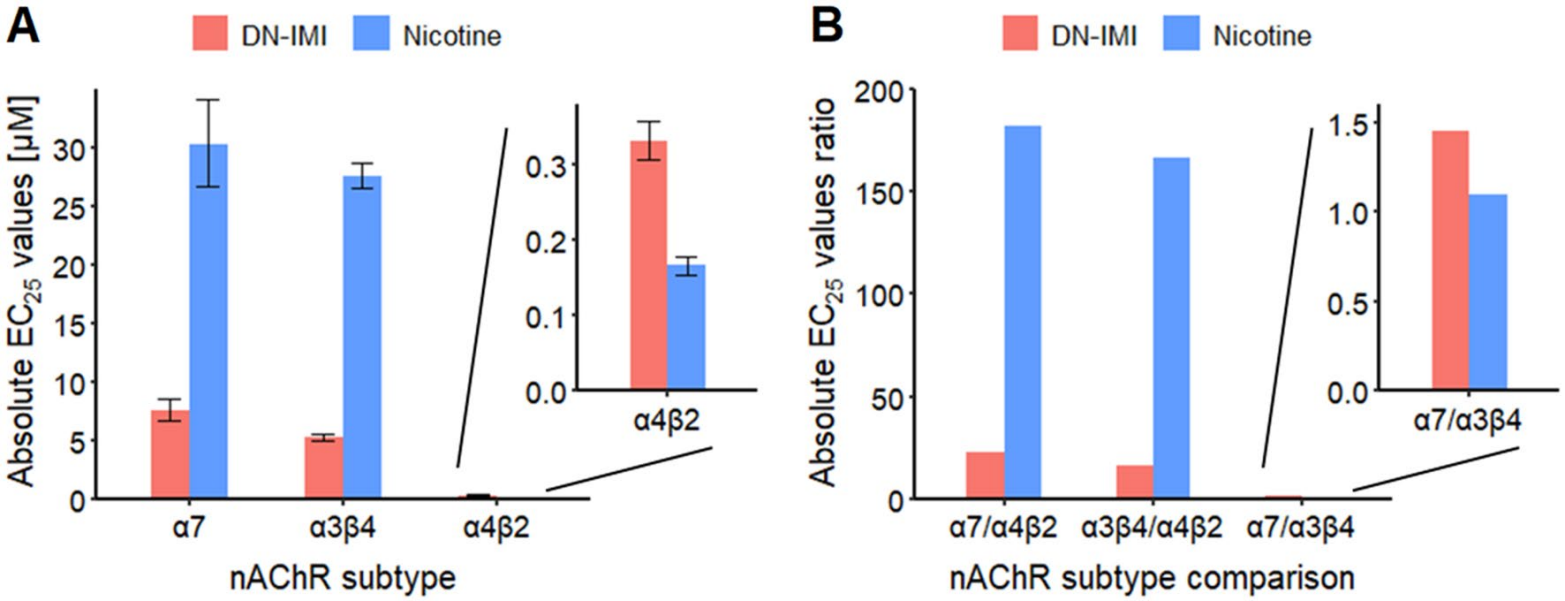
Comparative display of agonist potencies at nAChRs. Oocyte recordings were performed, and data for nicotine and DN-IMI stimulations are normalized as in Fig. 5. From the curve-fitted concentration–response data, EC_25_ values were determined. **A** The absolute EC_25_ values are shown for the effects on α7 (7.6 µM by DN-IMI and 30.2 µM by nicotine), α3β4 (5.2 µM by DN-IMI and 27.5 µM by nicotine), and α4β2 (HS) (0.33 µM for DN-IMI and 0.17 µM for nicotine) nAChRs. Note that the latter data set is shown as insert, because of the altered *y*-axis. **B** The ratios of the absolute EC_25_ values between the nAChR subtypes α7, α3β4, and α4β2 (HS) are displayed for the effects of DN-IMI and nicotine. HS = high-sensitivity variant of the receptor (two α4 subunits per receptor); note the different y-axis of the insert

Having obtained data on nicotine and DN-IMI for nAChR subtypes, we used them for a comparison of their potencies. For this purpose, we determined the absolute EC_25_ values (Fig. 6A). These data suggest that DN-IMI and nicotine were about equipotent on the α4β2 (HS) nAChR (less than half a log-step difference). On the other receptors, DN-IMI appeared slightly more potent than nicotine (about 0.6 log-steps). To understand differences between experimental systems or possibly to predict toxicological consequences for brain areas with different receptor expression patterns, it was interesting to compare apparent (functional) receptor affinities: this showed that both ligands were more potent on the α4β2 (HS) nAChR than on other subtypes (> 1 log-step for DN-IMI; > 2 log-steps for nicotine), while there was no difference between, e.g., α7 and α3β4 (Fig. 6B). This might explain mixed responses, e.g., on LUHMES cultures that express all these receptor types, and it provides an explanation for differences between, e.g., SH-SY5Y cells and LUHMES (the former cells predominantly express α7 receptors but also α3-containing receptors (Loser et al. 2021a)).

In summary, the metabolite DN-IMI exhibits significantly higher potency and efficacy on the human nAChR subtypes than its parent compound IMI. We performed extensive molecular docking studies of nicotine, IMI, DN-IMI, and IMI-olefin to further substantiate the experimental findings from oocytes and to provide a molecular explanation. The modeling results suggest a positioning of DN-IMI similar to that of nicotine at the binding sites of two nAChR subtypes. In contrast, IMI and IMI-olefin tend to adopt inverted and less favorable binding poses (Figs. S8 and S9). The docking studies thus provide a potential explanation for the lower signaling potency of these two compounds compared to DN-IMI.

### Exposure considerations and in vitro-to-in vivo comparisons

While the above approaches inform on potential hazards by DN-IMI, the interpretation of the data and their use for risk assessment requires some understanding of concentrations to be reached in human tissues/body fluids. Due to the lack of more direct human data, we built a physiology-based toxicokinetic (PBTK) model to predict the plasma concentrations of DN-IMI. Because of the limited availability of human metabolism and exposure data for DN-IMI, the model construction was based on data from atenolol, a compound with similar physicochemical properties, and with well-known human pharmacokinetics. As DN-IMI-specific parametrization of the model, we used metabolic turnover data from human primary hepatocytes and physicochemical properties of DN-IMI as predictors for passive membrane permeability and protein binding (Table S8). As input (oral exposure), we used 0.016 mg DN-IMI/kg body weight. This amount corresponds to 10% of the value used earlier for IMI PBTK modeling (Loser et al. 2021a). Our rationale was that DN-IMI can reach about 10% of the IMI content in fruits, vegetables, and cereals (see the introduction for reference).

Under these conditions, the model predicted average plasma concentrations of around 50 nM and peak concentrations in a subfraction of the human population of at least 100 nM (Fig. S10A). The plasma concentrations predicted for atenolol from our PBTK model were in good agreement with measured data found in the literature (Fig. S10B). We see this as an indication of a good predictive capacity of our model. As the central nervous system is a main target tissue of DN-IMI, we also predicted brain concentrations. They were even slightly higher than the plasma concentrations (Fig. S10C). It is likely that the free diffusion of the compound through the blood–brain barrier also predicts a free distribution into the fetus. It is, therefore, reasonable to assume that also fetal brains would be exposed to DN-IMI at concentrations up to the three-digit nM range.

BMC modeling of our [Ca^2+^]_i_ signaling and single nAChR data showed 20% response (in different systems) at about 100–300 nM of DN-IMI (Tables S9 and S10). Such concentrations are close to the ones reachable in some subjects by dietary exposure. While such concentrations may not be reached for the average of the population, the gap between realistic internal exposure levels and the minimal effect concentration is less than tenfold. This marginal safety buffer is eliminated, if receptor desensitization is considered as an effect parameter: the BMC for this endpoint was at ~ 17 nM (Table S11, for a 20% effect). Such concentrations may be reached by the consumption of food derived from crops treated with IMI. Notably, the desensitizing effect may be equally problematic for normal brain function and neuronal development, as the direct activation of the nAChRs.

To conclude these preliminary risk assessment considerations, it is important to consider that exposure to DN-IMI may also occur through the metabolism of IMI after it has been ingested. From rodent experiments, it is clear that DN-IMI is generated after exposure to IMI, and that the endogenous metabolite DN-IMI distributes to the brain (Chao and Casida 1997; Ford and Casida 2006). In addition, goat data suggest that IMI is converted to DN-IMI (about 25% of the IMI dose recovered in the liver) (Anon 2006). In rabbits, DN-IMI was excreted in the urine after exposure to IMI (Vardavas et al. 2018), and this agrees well with human biomonitoring data that identified high (several fold higher than IMI) levels of DN-IMI in urine (Wang et al. 2020).

If one assumes that 10% of ingested IMI is converted to DN-IMI, then the endogenously formed metabolite may reach levels of a similar magnitude as those generated from direct ingestion of the metabolite (assuming that the intake of IMI is 10 times higher than that of DN-IMI (input parameter of our PBTK model, based on food consumption data)). Therefore, a mixed exposure to IMI, DN-IMI, but also other metabolites, either produced endogenously (see PBTK model) or exogenously (see introduction), seems to be realistic and may lead to the summation of their adverse effects on the organism.

Even though such considerations of potential internal exposure are consistent with the literature data, they need to be considered as very preliminary. There is still considerable uncertainty on the human metabolism. It is not known which percentage of IMI is metabolized to DN-IMI within the liver and whether other tissues also contribute to the metabolism. The situation is complex, as several competing enzymes may oxidize or reduce IMI. Besides cytochrome P450 enzymes, there is evidence for the contribution of cytosolic aldehyde oxidases (Dick et al. 2005; Swenson and Casida 2013; Vardavas et al. 2018). These enzymes show high species variation in their expression and activity (Dick et al. 2005; Pryde et al. 2010). Considering that humans express relatively high levels of aldehyde oxidase, data from animals cannot be easily translated to humans, and experiments are ongoing to better quantify IMI metabolism by different cell compartments.

## Conclusions and outlook

The present study shows that the IMI metabolite DN-IMI potently (at sub-micromolar concentrations) affects human nAChRs. This was found both in neuronal cultures and in defined individual receptor subtypes expressed in *Xenopus laevis* oocytes. The evidence from all systems clearly indicates a much higher potency of DN-IMI relative to its parent compound IMI. The comparative data show that the desnitro metabolite is equipotent to nicotine, while another IMI metabolite, IMI-olefin, rather was equipotent to IMI. The study on DN-IMI showcases the role of metabolism for human neurotoxicology, as it demonstrates that a particular metabolite can be several orders of magnitude more potent as a neuronal signaling disrupter (desensitization) than its parent compound. This may have consequences for the risk assessment of the parent compound and for the need of additional data on metabolite generation in the environment and in man. Our preliminary modeling suggests that bioactive, potentially toxic DN-IMI concentrations may be reached by nutritional exposure in the normal (not professionally exposed) population.

Median lethal dose (LD_50_) studies with mice showed that DN-IMI (LD_50_: 6–24 mg/kg) is more toxic than its parent compound IMI (LD_50_: 35–50 mg/kg) (Chao and Casida 1997; Tomizawa et al. 2000, 2001) and IMI-olefin (no lethality at the highest tested dose of 50 mg/kg) (Chao and Casida 1997). Little information is available on more subtle forms of neurotoxicity, and to our knowledge, no data are available on the potential developmental neurotoxicity of DN-IMI. The latter is important, considering that nicotine is an established developmental neurotoxicant (Levin et al. 1993; LeSage et al. 2006; Aschner et al. 2017). The former is relevant, as different nAChR subtypes are present on, e.g., dopaminergic neurons and play an important role in the modulation of the electrical activity and neurotransmitter release (Rapier et al. 1988; Grady et al. 1992; Quik and Kulak 2002; Mameli-Engvall et al. 2006; Quik and Wonnacott 2011; de Kloet et al. 2015). Thus, substance-induced disturbances of nicotinic signaling can have an impact on the functioning, plasticity, and development of the nervous system (Wheeler and Cooper 2004; Welsby et al. 2006; Slotkin et al. 2006; Ziviani et al. 2011; Lozada et al. 2012; de Kloet et al. 2015; Romoli et al. 2019).

Several studies reported that the binding affinity of DN-IMI to mammalian or chicken nAChRs was similar to the affinity of nicotine and clearly higher than the one of its parent compound IMI (Chao and Casida 1997; Tomizawa and Casida 1999, 2000; D’Amour and Casida 1999; Tomizawa et al. 2000). Our functional data using a physiological signaling response in human neurons are in line with these observations. DN-IMI triggered [Ca^2+^]_i_ responses at concentrations ≥ 100 nM, i.e., it was at least two orders of magnitude more potent than its parent compound (Loser et al. 2021a). These findings were further supported by oocyte recordings, which showed an agonistic effect of DN-IMI on human α7 and several non-α7 nAChRs. DN-IMI activated α4β2 (HS) receptors at 20-fold lower concentrations than α7 and α3β4 nAChRs. This potency difference on α7 and α3β4 vs α4β2 (HS) is also seen for nicotine. Such relative receptor preferences may be responsible for a selective toxicity on certain brain regions or neuronal functions, and future studies should also include assays on non-neuronal nAChR.

In desensitization experiments with LUHMES, the pretreatment with DN-IMI inhibited the subsequent activation of the nAChRs at concentrations ≥ 10 nM (BMR10, Table S11), which is ~ 70 times more potent compared to the effects of IMI (Loser et al. 2021a). The more potent desensitization effect of DN-IMI in comparison with IMI was confirmed in SH-SY5Y neurons (Fig. S2). After prolonged agonist exposure, nicotinic receptors desensitize by adopting a high-affinity and agonist-bound, non-conducting conformation (Nemecz et al. 2016; Morales-Perez et al. 2016). This may adversely affect normal neuronal function and neurodevelopment.

For adverse outcome pathways (AOPs), it is important to understand the molecular initiating events (MIEs) both for parent compounds and also for the relevant metabolites formed (Leist et al. 2017). Until now, few such cases have been fully resolved, as the focus in neurotoxicology has either been on toxicants acting independent of metabolism, e.g., rotenone or vinca alkaloids (Delp et al. 2018b, 2021), or on compounds that act by a single toxic metabolite, without any effect of the parent, such as methyl-phenylpyridinium (Schildknecht et al. 2015; Terron et al. 2018) or methylmercury (Aschner et al. 2017). In many other cases, the target is little defined (e.g., for solvents or acrylamide). In this context, mechanistic studies on neonicotinoids and their metabolites should eventually provide an explanation for different potencies and activity spectra of all metabolites on various nAChRs. While we provide here evidence for the stimulation of nAChRs and on the attenuation of signaling (by desensitization) as MIEs, it is not possible to predict the most relevant adverse outcome. The reason for this is that nicotinic receptors are widespread throughout the central nervous systems and they are crucial for a large panel of higher order nervous system functions (Levin et al. 2006; Gotti et al. 2006; Zoli et al. 2015; Terry and Callahan 2019).

Concerning the understanding of the MIE, we used molecular docking studies to provide a rationale for the experimental findings. The availability of several X-ray structures with co-crystallized neonicotinoids has facilitated the establishment of a robust docking model (Ihara et al. 2008, 2014; Loser et al. 2021a). In the present work, we focused on the overlap of the pesticide N-heteroaromatic ring with the pyridine ring of nicotine. The comparison with published studies showed good accuracy of our model. Structural alignments of these complexes and docking studies at human nAChRs demonstrate that the electronegative moiety in IMI can contribute to a flip of the imidazolidine ring in the binding pocket. We demonstrate here that this is less likely to happen with DN-IMI. This feature may explain its higher affinity/potency. Our binding hypothesis is supported by ranking via different docking scores, binding free energy approximates, and comparisons of nicotinoids and neonicotinoids bound to homologous proteins. This gives a rationale for the functional differences of neonicotinoids and nicotine that were reported for cell experiments with LUHMES and SH-SY5Y cells (Loser et al. 2021a). These studies are still mainly qualitative, and their applicability domain is most likely narrow (applying only to the compounds of this study). However, our approach forms the basis for the development of a more powerful and refined model in the future. Eventually, this might then be able to quantitatively predict MIEs for the dozens of neonicotinoid metabolites found in food. Such a model might distinguish, e.g., high- vs. low-affinity ligands or discriminate between agonists and antagonists.

Further research is also needed to elucidate whether the signaling disturbances revealed here have lasting effects on neuronal function. It has been reported that other nAChR agonists (including nicotine) may affect nervous system plasticity and development (Levin et al. 1993; Wheeler and Cooper 2004; Welsby et al. 2006; Slotkin et al. 2006; Ziviani et al. 2011; Lozada et al. 2012; Romoli et al. 2019). Epidemiological studies are quite scarce, but some general developmental/neurological effects have been reported for neonicotinoids used in agriculture or anti-tick sprays (Cimino et al. 2017).

Some other compounds that evoke disturbances of neuronal network activity without causing structural changes have been reported to induce developmental neurotoxicity (DNT). Examples are 3,4-methylenedioxymethamphetamine (MDMA, ecstasy), heroin, or nicotine (Levin et al. 1993; Slikker Jr et al. 2005; LeSage et al. 2006; Dwyer et al. 2009; Slotkin et al. 2016; Aschner et al. 2017). Moreover, compounds for example methylmercury and lead can have severe effects on the developing brain, although they have a low neurotoxicity for adults (Grandjean and Landrigan 2014). These examples make it conceivable that neonicotinoids and their metabolites such as DN-IMI may exhibit a DNT risk. However, a transfer of knowledge from one compound (e.g., nicotine) to others (e.g., DN-IMI) holds the risk of uncertainties (Rovida et al. 2020). Therefore, further mechanistic studies are needed to address the difficult question of a DNT hazard of DN-IMI and other neonicotinoid metabolites like a descyano metabolite of thiacloprid, which has also been reported to exhibit a higher affinity for mammalian and chicken nAChRs than its parent compound (Tomizawa and Casida 2000; Tomizawa et al. 2000).

## Supplementary Information

Below is the link to the electronic supplementary material.

Supplementary file1 (DOCX 2356 kb)

## Abbreviations

ACh: Acetylcholine
AUC: Area under the curve
BMC: Benchmark concentration
[Ca^2^^+^]_i_: Intracellular free Ca^2^^+^ concentration
cAMP: N6,2′-0-Dibutyryl 3′,5′-cyclic adenosine monophosphate
DN-IMI: Desnitro-imidacloprid
DNT: Developmental neurotoxicity
DMSO: Dimethyl sulfoxide
EC_25_: Quarter maximal effective concentration
IMI: Imidacloprid
IMI-olefin: Imidacloprid-olefin
LD_50_: Median lethal dose
Mec: Mecamylamine
MIE: Molecular initiating event
MLA: Methyllycaconitine
nAChR: Nicotinic acetylcholine receptor
pEC_25_: Negative logarithm of the quarter maximal effective concentration
pEC_50_: Negative logarithm of the half-maximal effective concentration
pIC_50_: Negative logarithm of the half-maximal inhibitory concentration
PBTK: Physiology-based toxicokinetic
PLO: Poly-l-ornithine
PNU: PNU-120596
Tubo: Tubocurarine
