# Supplementary material for: Acute effects of the imidacloprid metabolite desnitro-imidacloprid on human nACh receptors relevant for neuronal signaling
Source: Arch Toxicol. 2021 Oct 10;95(12):3695–716. doi: 10.1007/s00204-021-03168-z (PMC8536575; doi:10.1007/s00204-021-03168-z)
Supplement: Supplementary file 1 — Supplementary file1 (DOCX 2356 kb) [file 204_2021_3168_MOESM1_ESM.docx]

**Supplementary information for**

**Acute effects of the imidacloprid metabolite desnitro-imidacloprid on human nACh receptors relevant for neuronal signaling**

*Dominik Loser^1,2^, Karin Grillberger^3^, Maria G. Hinojosa^4^, Jonathan Blum^2^, Yves Haufe^5^,* *Timm Danker^1^, Ylva Johansson^4^, Clemens Möller^6^, Annette Nicke^5^*, *Susanne H. Bennekou^7^, Iain Gardner^8^, Caroline Bauch^9^, Paul Walker^9^, Anna Forsby^4^, Gerhard F. Ecker^3^, Udo Kraushaar^1,#^, Marcel Leist^2,#^*

^1^ NMI Natural and Medical Sciences Institute at the University of Tübingen, 72770 Reutlingen, Germany

^2^ In vitro Toxicology and Biomedicine, Dept inaugurated by the Doerenkamp-Zbinden foundation, University of Konstanz, 78457 Konstanz, Germany

^3^ Department of Pharmaceutical Chemistry, University of Vienna, Vienna, Austria

^4^ Department of Biochemistry and Biophysics, Stockholm University, 106 91 Stockholm, Sweden

^5^ Walther Straub Institute of Pharmacology and Toxicology, Faculty of Medicine, LMU Munich, 80336 Munich, Germany

^6^ Life Sciences Faculty, Albstadt-Sigmaringen University, 72488 Sigmaringen, Germany

^7^ Technical University of Denmark, Kongens Lyngby, Denmark

^8^ CERTARA UK Limited, Simcyp Division, Level 2-Acero, 1 Concourse Way, Sheffield, S1 2BJ, UK

^9^ Cyprotex Discovery Ltd, No. 24 Mereside, Alderley Park, Cheshire, SK10 4TG, UK

^#^ These authors contributed equally.

| **Table of Contents** | | |
| --- | --- | --- |
| **Fig. S1** | Activation of human α7 nAChRs on LUHMES neurons by IMI-olefin. | page 3 |
| **Fig. S2** | Modulation of cholinergic responses of SH-SY5Y cells by DN-IMI, IMI, and acetamiprid. | page 4 |
| **Fig. S3** | Effects of ACh, DN-IMI, and nicotine on human α4β2 (HS) nAChRs expressed by *Xenopus laevis* oocytes. | page 5 |
| **Fig. S4** | Inhibition of DN-IMI-induced current responses of human nAChRs expressed by *Xenopus laevis* oocytes. | page 6 |
| **Fig. S5** | Effects of DN-IMI, IMI-olefin, IMI, and nicotine on human α7 nAChRs expressed by *Xenopus laevis* oocytes. | page 7 |
| **Fig. S6** | Effects of ACh, DN-IMI, IMI, and nicotine on human α3β4 nAChRs expressed by *Xenopus laevis* oocytes. | page 8-9 |
| **Fig. S7** | Effects of DN-IMI and IMI on human α4β2 (LS) and α4β4 nAChRs expressed by *Xenopus laevis* oocytes*.* | page 10 |
|  | Molecular docking studies of nicotine, IMI, and IMI metabolites on nAChR models. | page 11-13 |
| **Fig. S8** | Molecular docking studies on nAChR structures. | page 13-14 |
| **Fig. S9** | Molecular docking studies. | page 15 |
|  | Materials and methods for molecular docking studies: Structure-based approach – docking studies and binding free energy calculations. | page 16-17 |
| **Fig. S10** | Physiologically-based toxicokinetic (PBTK) modeling of DN-IMI and atenolol in the human population. | page 18-19 |
| **Table S1** | Compound list. | page 20 |
| **Table S2** | Overview of pEC_50_ values for agonist experiments. | page 21 |
| **Table S3** | Overview of percentages of responsive cells of single-cell Ca^2+^-imaging. | page 21 |
| **Table S4** | Overview of pIC_50_ values. | page 22 |
| **Table S5** | Overview of pEC_50_ and maximum values for *Xenopus laevis* oocyte experiments. | page 22 |
| **Table S6** | Overview of concentrations and technical replicates. | page 23-25 |
| **Table S7** | IFD and MM-GBSA-energy calculation parameters. | page 26 |
| **Table S8** | Input parameters used in the DN-IMI and atenolol PBTK model. | page 27 |
| **Table S9** | Benchmark responses (BMR) for the [Ca^2+^]_i_ responses of LUHMES neurons triggered by DN-IMI and IMI-olefin. | page 28 |
| **Table S10** | Benchmark responses (BMR) for the agonistic effects of nicotine and DN-IMI on several human nAChR subtypes expressed in *Xenopus laevis* oocytes. | page 28 |
| **Table S11** | Benchmark responses (BMR) for the desensitizing effect of DN-IMI and IMI-olefin on nAChR signaling. | page 29 |
|  | References. | page 29-32 |


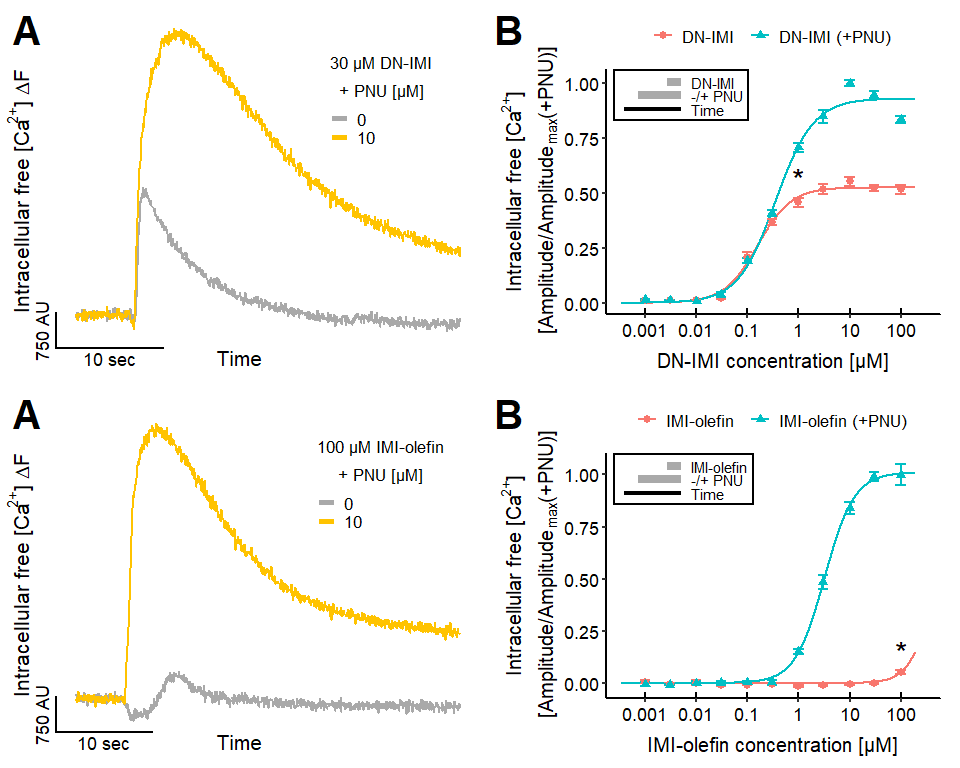


**Fig. S1: Activation of human** **α7 nAChRs on LUHMES neurons by IMI-olefin.**

LUHMES neurons were cultivated in 384-well plates and used for Ca^2+^-imaging. **(A)** The cells were exposed to various concentrations of IMI-olefin in the absence and presence of PNU-120596 (PNU, 10 µM), and exemplary recordings of the fluorescence signal from a whole well are shown. **(B)** The fluorescence data (peak amplitude) of multiple experiments were quantified and normalized to the maximal response triggered by IMI-olefin in the presence of PNU (means ± SEM are displayed), and after curve fitting a pEC_50_ value of 5.5 ± 0.03 was determined for IMI-olefin in the presence of PNU. The significance of the responses triggered by IMI-olefin was evaluated between the responses evoked by IMI-olefin in the absence and presence of PNU (*: p < 0.05). Note the treatment scheme (upper left corner), illustrating the experimental design. Detailed data on n numbers are found in table S6.


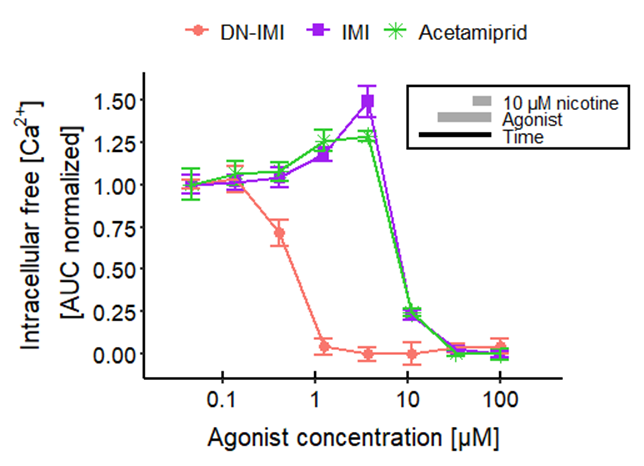


**Fig. S2: Modulation of cholinergic responses of SH-SY5Y cells by DN-IMI, IMI, and acetamiprid.**

SH-SY5Y cells were pretreated with various concentrations of DN-IMI, IMI, and acetamiprid for 20 min, before nicotine (10 µM) was applied in the presence of PNU-120596 (PNU, 10 µM) and the [Ca^2+^]_i_ response was recorded. The area under the curve (AUC) was determined for the fluorescence intensity of the responses triggered by nicotine. The data were normalized to the [Ca^2+^]_i_ responses stimulated by nicotine in the presence of the lowest agonist concentration. No curve fitting was performed. Note the treatment scheme (upper right corner), illustrating the experimental design.


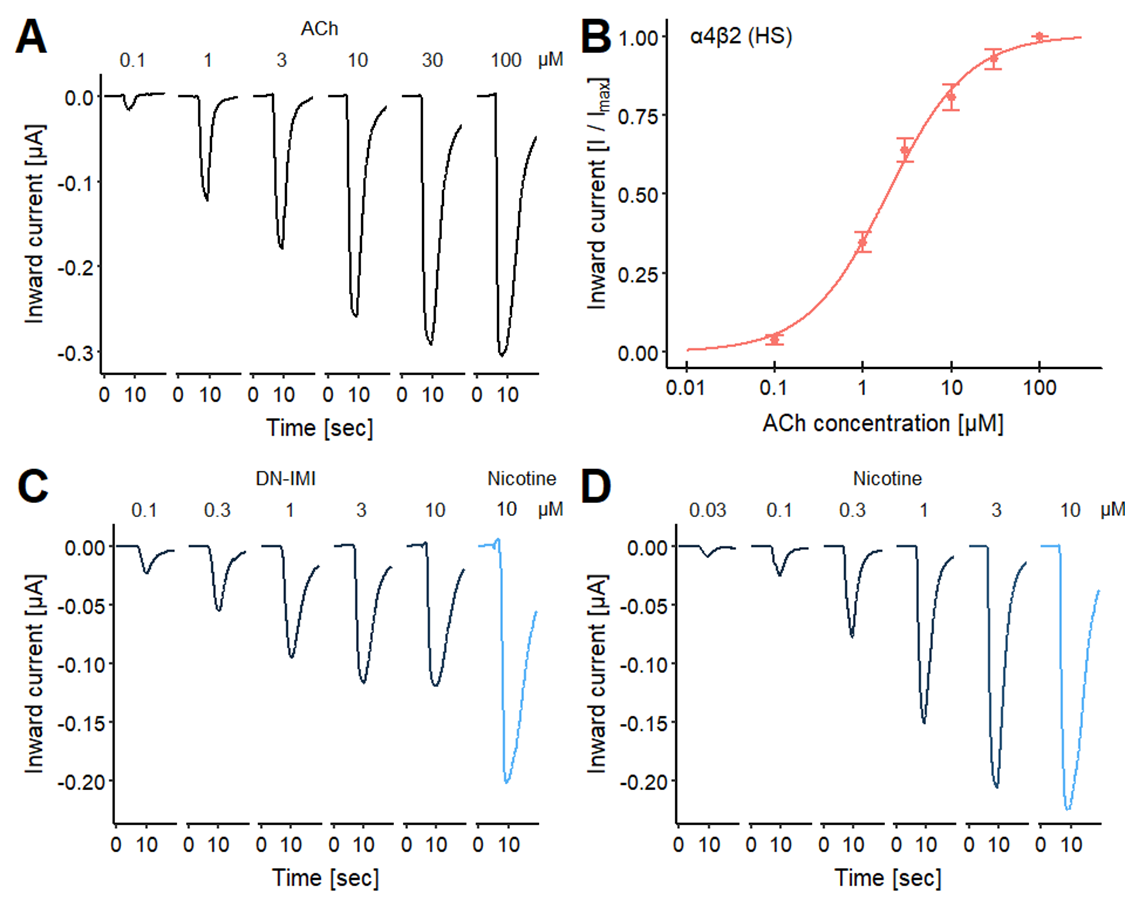


**Fig. S3: Effects of ACh, DN-IMI, and nicotine on human α4β2 (HS) nAChRs heterologously expressed by *Xenopus laevis* oocytes.**

**(A)** Exemplary inward currents through human α4β2 (HS) nAChRs heterologously expressed by *Xenopus laevis* oocytes stimulated by various ACh concentrations are shown. **(B)** The inward current data (amplitude) of human α4β2 (HS) nAChRs heterologously expressed by *Xenopus laevis* oocytes of multiple experiments were quantified (means ± SEM are displayed), and after curve fitting a pEC_50_ value of 5.7 ± 0.06 for ACh was determined. **(C, D)** Exemplary inward currents through human α4β2 (HS) nAChRs heterologously expressed by *Xenopus laevis* oocytes triggered by various concentrations of **(C)** DN-IMI and **(D)** nicotine are shown together with the response to a final reference application of nicotine (10 µM, reference response (blue)).


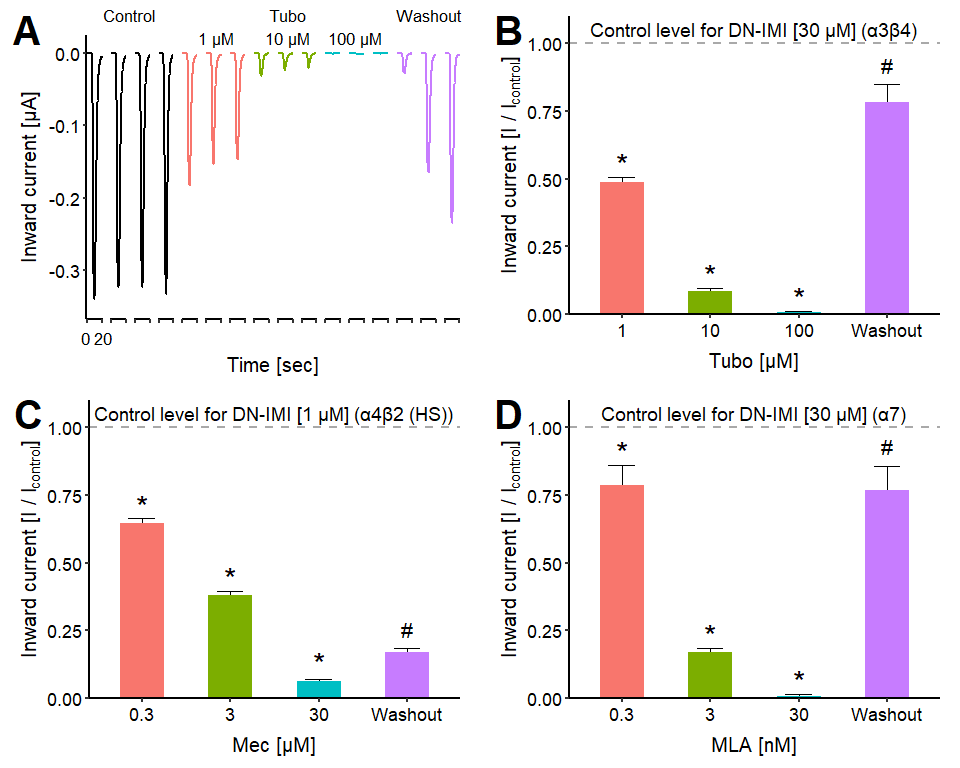


**Fig. S4: Inhibition of DN-IMI-induced current responses of human nAChRs heterologously expressed by Xenopus laevis oocytes.**

**(A)** Exemplary traces of inward currents of the human α3β4 nAChR triggered by DN-IMI (30 µM) during control, in the presence of different concentrations of the nAChR antagonist Tubo, and during washout are shown. **(B)** The inhibitory effect of Tubo on the inward current amplitude of the human α3β4 nAChR evoked by DN-IMI (30 µM) was evaluated. **(C)** The inhibitory effect of the non-competitive nAChR antagonist Mec on the inward current amplitude of the human α4β2 (HS) nAChR triggered by DN-IMI (1 µM) was determined. **(D)** The antagonistic effect of the potent α7 nAChR blocker MLA on the inward current amplitude of the human α7 nAChR stimulated by DN-IMI (30 µM) was evaluated. The significance of the inward current block was determined between control recordings and recordings in the presence of the antagonist (*: p < 0.05). The significance of the inward current increase after washout of the antagonist was evaluated between recordings of the highest antagonist concentration and recordings during washout (#: p < 0.05). Detailed data on n numbers are found in table S6.


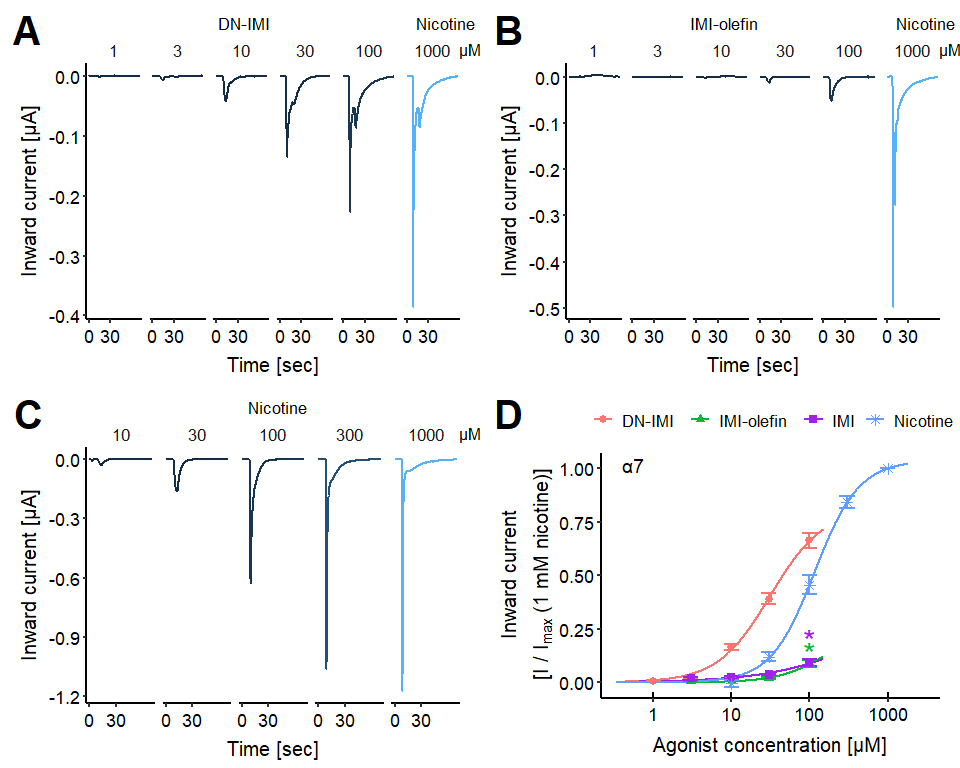


**Fig. S5: Effects of DN-IMI, IMI-olefin, IMI, and nicotine on human α7 nAChRs heterologously expressed by *Xenopus laevis* oocytes.**

**(A-C)** Exemplary inward currents through human α7 nAChRs heterologously expressed by *Xenopus laevis* oocytes stimulated by various concentrations of **(A)** DN-IMI, **(B)** IMI-olefin, and **(C)** nicotine are shown together with the response to a final reference application of nicotine (1 mM, reference response (blue)). **(D)** The inward current data (amplitude) of human α7 nAChRs heterologously expressed by *Xenopus laevis* oocytes of multiple experiments were quantified (means ± SEM are displayed), and after curve fitting relative pEC_50_ values of 4.5 ± 0.09 (estimated maximum ~0.81) for DN-IMI and 3.9 ± 0.04 (estimated maximum ~1) for nicotine were determined. The significance of the responses triggered by IMI-olefin and IMI was evaluated between the lowest concentration (3 µM) and the other concentrations (*: p < 0.05). The current amplitudes were normalized to the response induced by nicotine (1 mM) for each oocyte. Detailed data on n numbers are found in table S6.


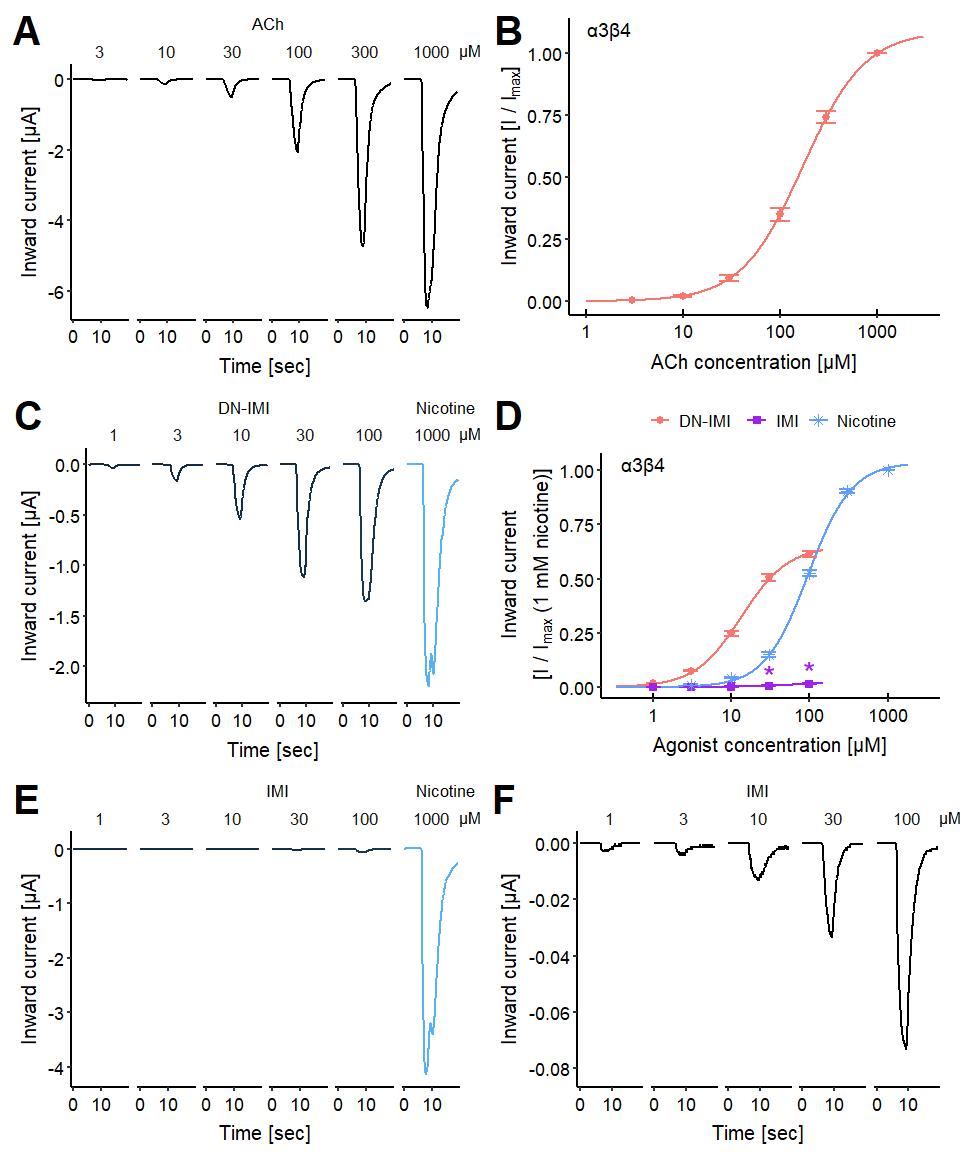


**Fig. S6: Effects of ACh, DN-IMI, IMI, and nicotine on human α3β4 nAChRs heterologously expressed by *Xenopus laevis* oocytes.**

**(A)** Exemplary inward currents through human α3β4 nAChRs heterologously expressed by *Xenopus laevis* oocytes stimulated by various ACh concentrations are presented. **(B)** The inward current data (amplitude) of human α3β4 nAChRs heterologously expressed by *Xenopus laevis* oocytes of multiple experiments were quantified (means ± SEM are displayed), and after curve fitting a relative pEC_50_ value of 3.8 ± 0.03 for ACh was determined. **(C)** Exemplary inward currents through human α3β4 nAChRs heterologously expressed by *Xenopus laevis* oocytes triggered by various concentrations of DN-IMI are shown together with the response to a final reference application of nicotine (1 mM, reference response (blue)). **(D)** The inward current data (amplitude) of human α3β4 nAChRs heterologously expressed by *Xenopus laevis* oocytes of multiple experiments were quantified (means ± SEM are displayed), and after curve fitting relative pEC_50_ values of 4.9 ± 0.03 (estimated maximum ~0.65) for DN-IMI and 4.0 ± 0.01 (estimated maximum ~1) for nicotine were determined. The significance of the responses triggered by IMI was evaluated between the lowest concentration (1 µM) and the other concentrations (*: p < 0.05). The current amplitudes were normalized to the response induced by nicotine (1 mM) for each oocyte. **(E, F)** Exemplary inward currents through human α3β4 nAChRs heterologously expressed by *Xenopus laevis* oocytes triggered by various concentrations of IMI are shown with a y-axis adjusted to **(E)** the amplitude of the response to a final reference application of nicotine (1 mM, reference response (blue)) and **(F)** the highest current amplitude stimulated by IMI (100 µM). Detailed data on n numbers are found in table S6.


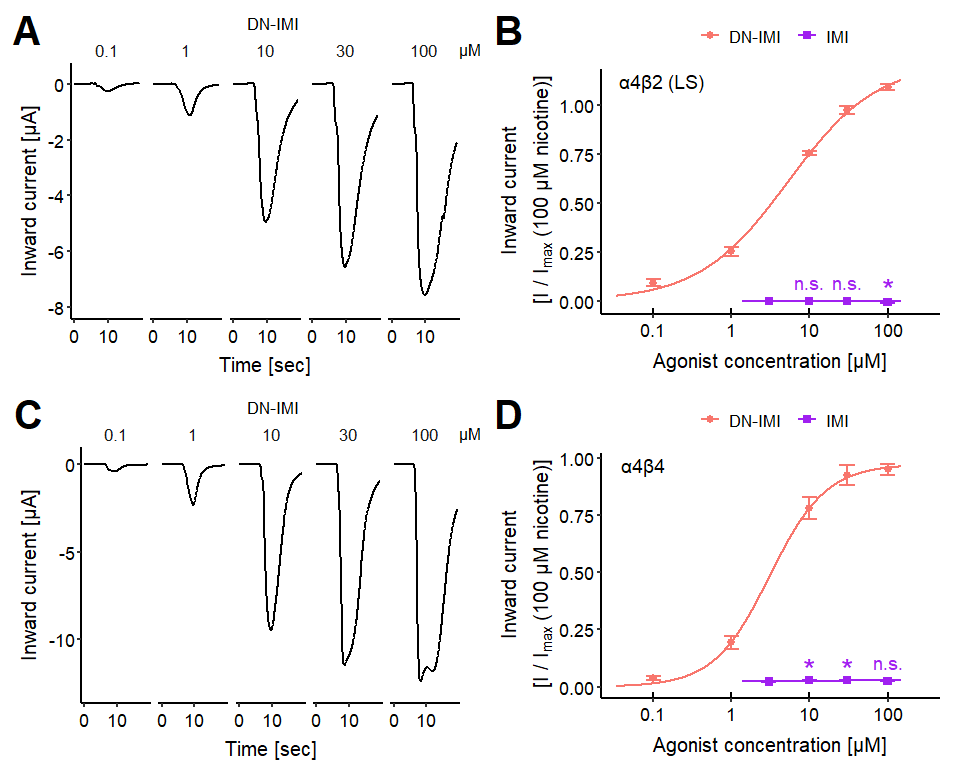


**Fig. S7: Effects of DN-IMI and IMI on human α4β2 (LS) and α4β4 nAChRs heterologously expressed by Xenopus laevis oocytes.**

**(A)** Exemplary inward currents through human α4β2 (LS) nAChRs heterologously expressed by *Xenopus laevis* oocytes stimulated by various DN-IMI concentrations are shown. **(B)** The inward current data (amplitude) of human α4β2 (LS) nAChRs heterologously expressed by *Xenopus laevis* oocytes of multiple experiments were quantified (means ± SEM are displayed), and after curve fitting a relative pEC_50_ value of 5.3 ± 0.06 (estimated maximum ~1.22) for DN-IMI was determined. The significance of the responses triggered by IMI was evaluated between the lowest concentration (3 µM) and the other concentrations (*: p < 0.05; n.s., not significant). The current amplitudes were normalized to the response induced by nicotine (100 µM) for each oocyte. **(C)** Exemplary inward currents through human α4β4 nAChRs heterologously expressed by *Xenopus laevis* oocytes stimulated by various DN-IMI concentrations are shown. **(D)** The inward current data (amplitude) of human α4β4 nAChRs heterologously expressed by *Xenopus laevis* oocytes of multiple experiments were quantified (means ± SEM are displayed), and after curve fitting a relative pEC_50_ value of 5.5 ± 0.07 (estimated maximum ~0.97) for DN-IMI was determined. The significance of the responses triggered by IMI was evaluated between the lowest concentration (3 µM) and the other concentrations (*: p < 0.05; n.s., not significant). The current amplitudes were normalized to the response induced by nicotine (100 µM) for each oocyte. Detailed data on n numbers are found in table S6.

**Molecular docking studies of nicotine, IMI, and IMI metabolites on nAChR models**

We were interested in how the particularly high potency of DN-IMI (as compared to other neonicotinoids) may be explained on the level of receptor binding. For this reason, we used the extensive data on nAChR structures to build models for molecular docking.

Ligand binding sites of nAChRs are positioned at the interface of an alpha subunit (principal or + face) with the complementary or - face of another, adjacent subunit. Thus, a typical binding site is found at the interface of an α4 subunit and a β2 subunit (Fig. S8A), or between two α7 subunits. A more detailed characterization of the ligand-binding site showed that it is formed by six polypeptide loops (designated A-F) (Karlin 2002). The amino acid side chains from these loops are forming a tightly packed “aromatic box” surrounding the agonist (Fig. S8B) (Morales-Perez et al. 2016). A conspicuous structural element is loop C, which is stabilized by a disulfide bond, and winds around the ligand (Fig. S8C, D) (Karlin 2002; Morales-Perez et al. 2016). It undergoes ligand-induced conformational changes and contributes to steric constraints (Li et al. 2011b). Data on co-crystallized complexes (nAChR with ligands) suggest that several neonicotinoids occupy the upper (extracellular-oriented) part of the ligand-binding pocket in an essentially similar fashion as nicotine. This means that the heteroarylic moiety overlaps with the position of nicotine’s pyridine ring. However, the nitroguanidine substructure occupies space that would normally be covered by loop C. This feature was very well reproduced by our dynamic docking model (Fig. S8B). In this binding mode, the electronegative nitro- or cyano-groups are pointing towards the tip of loop C of the principal subunit, while the arylic moieties are pointing towards loop E from the complementary subunit. DN-IMI is lacking the electronegative and space-requiring nitro-functionality. Instead, it features a positively chargeable guanidine-functionality, embedded in the imidazolidine ring (Fig. S8B). Our docking experiments at human nAChR structures suggest that this feature results in a flip of the imidazolidine-ring (relative to IMI), and therefore a much better alignment with the most likely poses of nicotine (Fig. S8C).

To obtain information on binding pockets from other nAChR subtypes, we used structural data on ACh binding protein (AChBP) from *Lymnaea stagnalis*. Good X-ray structures for this protein, also with several co-crystallized ligands are available and it is commonly accepted that this protein is a good model for mammalian α7 receptors (Ihara et al. 2008). Also here, neonicotinoids with an N-heteroaromatic ring (e.g., IMI) assume a binding mode that positions this structure in a very similar way as the pyridine ring of nicotine, while the nitroguanidine group faces loop C. Similar to the binding pocket of the α4β2 receptor, the docking suggested a much more favorable position for DN-IMI, with the aminoguanidine group facing away from loop C (Fig. S8D).

Molecular docking studies of such a complex target as the nAChR are not yet able to predict absolute binding affinities. However, there are several approaches to compare potential ligands. One such strategy is to calculate the ensemble of all likely docking poses for a ligand and to compare them with an optimal pose or with the exact positioning of a known ligand. For instance, it has been suggested that neonicotinoids may bind at mammalian receptors in the “common” mode or in a so-called “inverted” mode (pyridine moiety facing away from the central pore). In this inverted binding mode, the electronegative functionalities are located at the place of the N-heteroaromatic moieties in the common mode (Fig. S9B, C), and this appears energetically less favored (Tomizawa et al. 2008). Docking experiments on various receptor models suggested that the least potent neonicotinoids have the highest propensity to bind in the inverted mode (Loser et al. 2021). Our docking experiments suggested that DN-IMI prefers the common binding mode compared to the inverted position. This preferential behavior is also seen for nicotine (Fig. S8B). Docking scores were also obtained for IMI-olefin. The results suggest a similar behavior as that of IMI, i.e., there was a good fit with the binding site (in line with clear signaling data in LUHMES cells), but a predicted interference with loop C, showing less favorable contacts to amino acids from the aromatic box, which is also reflected in the worsened docking score and MM-GBSA binding energy approximate (Table S7) (in line with a lower potency than the one of DN-IMI and nicotine). The docking studies showed that the imidazolidine ring of IMI behaves similar to the imidazole ring (double bond between carbon atoms C4 and C5) of IMI-olefin. (Fig. S9D).

Besides the scoring of docking poses, some semi-quantitative data may be obtained by the calculation of proxies for binding energies. We evaluated via docking experiments and subsequent binding energy (MM-GBSA) calculations whether the inverted binding mode (more likely for IMI) shows differences in calculated binding energy compared to the common binding mode (more likely for DN-IMI and nicotine) (Fig. S9B, C). Indeed, the common binding mode ranked better (according to the ”MM-GBSA dG bind” parameter). In addition, our approach showed for the α4β2 model that IMI scored worse in the binding pose ranking than IMI-olefin. Moreover, the highest binding energy was predicted for DN-IMI (Table S7). However, some inconsistencies concerning nicotine still need to be explained. But this would require applying methods such as linear free energy calculations, which are much more time and computational cost-intensive than state-of-the-art docking approaches. As an alternative experimental approach to obtain data from individual specified receptors, we performed recordings in the *Xenopus laevis* oocyte expression system.


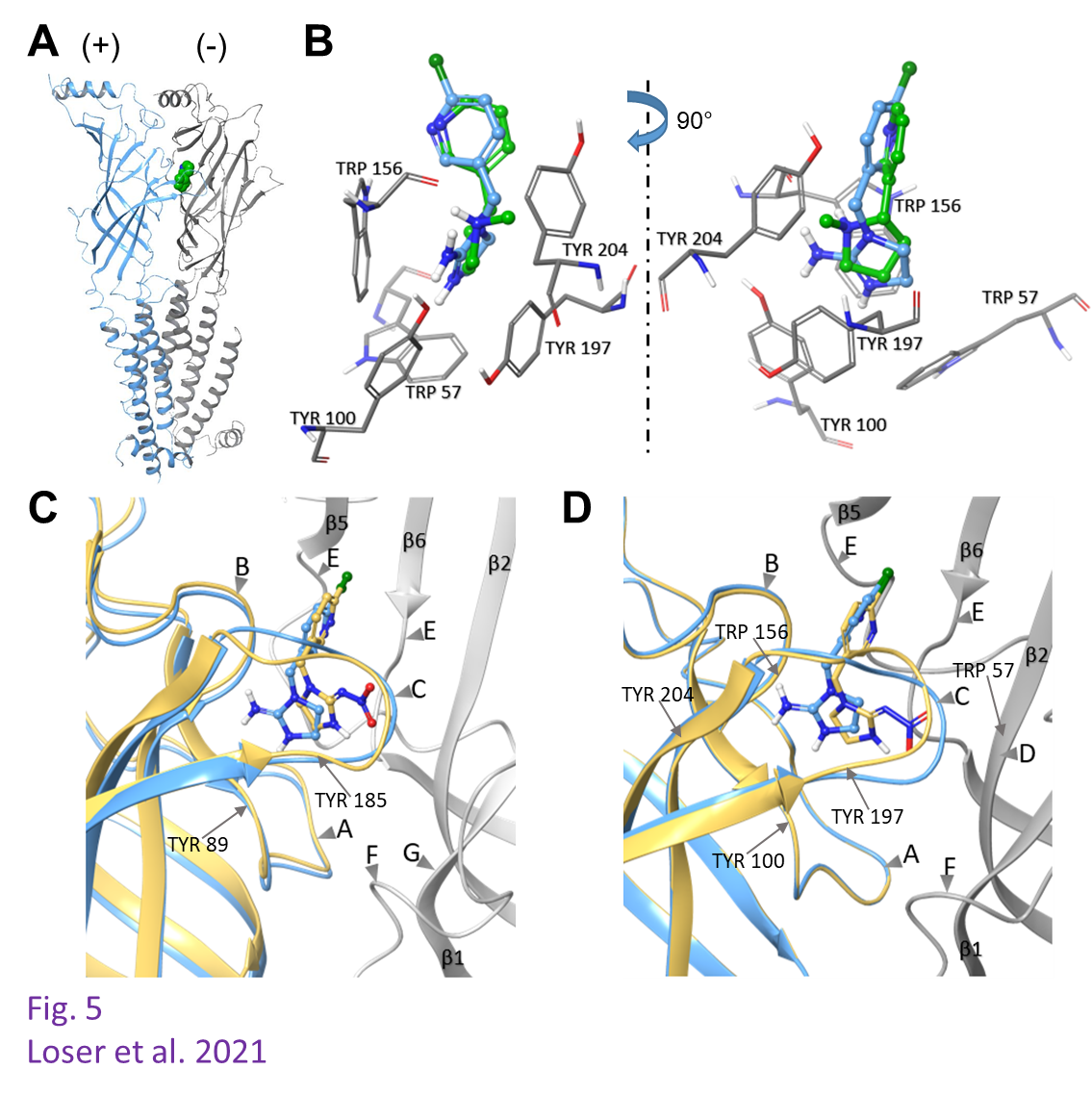


**Fig. S8: Molecular docking studies on nAChR structures.**

**(A)** A 3D model of the human pentameric α4β2 nAChR in its ligand-bound state was established. A side view (top: extracellular part; bottom: intracellular part) of two of the five receptor subunits is shown. Nicotine (green) is shown at its binding site (based on legacy data of co-crystallization studies) between an α subunit (+; blue ribbon) and a β subunit (-; grey ribbon). **(B)** Details are shown of the nicotine binding site, with particular focus on the amino acids of the “aromatic box”. Nicotine (green carbon atoms, ball-stick model) and DN-IMI (blue carbon atoms) are docked to the same site. Two view angles (turned by 90 degrees) are provided to show the close similarities in positioning and space requirements of the ligands. The amino features of both ligands are framed by residues from the aromatic box (a conserved protein structure amongst nAChRs). The canonical nomenclature of nAChR peptide backbone loops (shown in C, D) is used here for the localization of particular amino acids. Displayed amino acids from the principal binding site (α subunit) are Tyr100 (on loop A), Trp156 (on loop B), Tyr197, and Tyr204 (on loop C ). From the complementary binding site loop D (β subunit), Trp57 is shown. Note that DN-IMI is adopting a binding mode that resembles the one of nicotine, concerning both, the orientation of the arylic moiety as well as the amino feature. **(C)** IMI (yellow carbon atoms; blue nitrogen- and red oxygen atoms) and DN-IMI (blue carbon atoms) in complex with the human nAChR α4β2-structure. The structures were aligned based on the Cα-atoms. Note that the nitro-imidazolidine ring of IMI, with its nitro group, is facing towards the loop C (different from DN-IMI), while the pyridine rings of DN-IMI and IMI align well in their representative poses. IMI possibly interacts with Glu198 from loop C, and this interaction may reduce the affinity by steric hindrance and electrostatic repulsion. **(D)** Data on AChBP have been used to model α7 nAChRs (as is common in the field). Data on IMI (yellow carbon atoms) and DN-IMI (blue carbon atoms), co-crystallized with AChBP were extracted from a structure database. The ligands were superimposed here, based on the Cα-atoms. The protein ribbons of the principal side are shown in blue and yellow for the two structures used. The ribbon of one complementary face of the binding site is shown in light gray for a better view. Note that a very similar difference in binding poses can be noted for the α7 model (AChBP-based) as for the α4β2 nAChR model (in C).


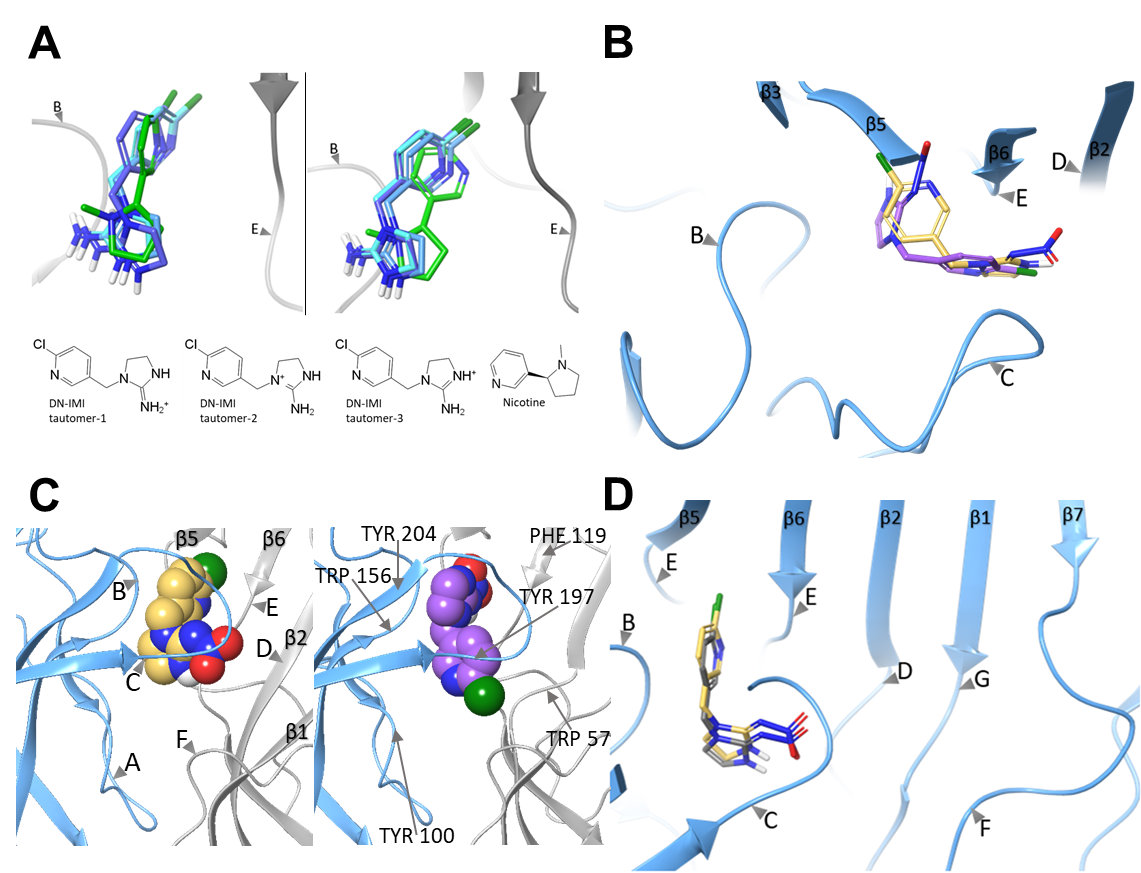


**Fig. S9: Molecular docking studies.**

**(A)** The IFD poses of DN-IMI in human α4β2 nAChR (grey ribbon) before (top left) and after (top right) the MMG-BSA incorporated minimization step are displayed, together with the chemical structures of the presented DN-IMI tautomers (at the bottom from left to right: tautomer 1 (turquoise), tautomer 2 (medium blue), tautomer 3 (dark blue)) and nicotine (bottom right, green). The different tautomer states of the guanidine moiety embedded in the imidazolidine ring and their effect on the docking score, MMG-BSA energy, and binding orientation in α4(+)β2(-)-binding site were investigated (Table S7). After the MMG-BSA applications, some torsion angles were optimized for a better alignment of the DN-IMI poses. **(B)** IMI-olefin is shown in the common (yellow) and inverted (purple) binding mode in the binding pocket of the human α7 nAChR (blue ribbon). **(C)** IMI is displayed in the common (yellow) and inverted (purple) binding mode in the binding pocket of the human α4β2 nAChR. The ribbon of one complementary face of the binding site is shown in light gray for a better view. **(D)** IMI (yellow) and IMI-olefin (gray) bind in the common binding mode to the structure of the human α4β2 nAChR (blue ribbon). MMG-BSA binding energy calculations predicted the inverted binding mode of IMI to be less favorable for the overall thermodynamic state of the protein-ligand complex compared to the common binding mode in the α4β2- and α7-binding site (Table S7).

**Materials and methods for molecular docking studies: Structure-based approach – docking studies and binding free energy calculations.**

In order to gather more information about the binding mode of nicotinoids (DN-IMI) and neonicotinoids (IMI) on human nAChRs, with the focus on the α7- and α4β2-isoforms, we also searched the protein data bank (PDB, rcsb.org) and relevant literature for accessible structures.

IMI and DN-IMI are available in complex of the soluble acetylcholine binding protein (AChBP) (PDB-ID: 2zju (Ihara et al. 2008) and 3wtn (Ihara et al. 2014)), which is homologous to the extracellular domain of nAChRs.

For the structure-based approach, we conducted induced-fit docking (IFD) experiments and binding energy calculations by using respective tools from the software suite of Schrödinger’s Maestro20-2 (Schrödinger Release 2020-2 2020). A detailed description of the IFD can be found in the methods section of our previous publication about neonicotinoid effects on human neurons (Loser et al. 2021). In brief, before the docking studies, the proteins and ligands of interest need to be prepared to enrich the quality of the docking outcome (Madhavi Sastry et al. 2013). Both have been prepared at pH 7.0 ± 0.5 using LigPrep and Protein Preparation Wizard, respectively, and additionally have been checked manually (Schrödinger Release 2020-2 2020). Nicotine has been structurally elucidated in complex of the human α4β2-isoform at distinct stoichiometries, so that PDB-ID 6cnj suited the purpose for the α4(+)β2(-)- and 6cnk for the α4(+)α4(-)-binding pocket (Walsh et al. 2018)**. The co-crystallized ligand nicotine between chains A and B,** [A:402] in 6cnj and [A:405] in 6cnk (Walsh et al. 2018), has been used as a centroid for the docking grid, respectively. Before the structure of the human nAChR α7-isoform has recently been experimentally resolved (Noviello et al. 2021), published homology models of the ligand-binding domain were usually used for docking studies on this subtype (Ng et al. 2018). The centroid of previously defined key ligand-binding residues (Ng et al. 2018; Sakkiah et al. 2020) has been used as the center of the grid box on this isoform.

**The docking poses are scored by the docking algorithm which implements the OPLS3e force field** (Harder et al. 2016; Roos et al. 2019)**, according to their estimated binding energy of the generated protein-ligand complex** (Schrödinger Release 2020-2 2020)**: Scoring** parameters from log-file: (1) IFDScore [kcal/mol] = + 1.0* Prime_Energy + 9.057 Glide_gscore + 1.428 Glide_ecoul; (2) docking Score [kcal/mol] = GlideScore + Epik State Penalty (from LigPrep). The Prime technology is not only applied during the IFD but is also used for calculations of the ligand binding energy with the molecular mechanics generalized Born surface area (MM-GBSA)-tool within the Schrödinger package (Schrödinger Release 2020-2: Prime, Schrödinger, LLC, New York, NY, 2020 (Schrödinger Release 2020-2 2020)). This method utilizes molecular mechanics energies in combination with a generalized Born model to assess residue-dependent effects and electrostatic solvation energy (Fig. S9A). Within the VSGB solvation model used in this study (Li et al. 2011a), water is set as a solvent that is suitable for the ligand-binding domain of nAChRs since it is located extracellularly and is, therefore, water exposed (Velisetty et al. 2014).

Also, surface areas have been incorporated in this binding energy assessment tool (Genheden and Ryde 2015; Schrödinger Release 2020-2 2020). The force field for refinement, OPLS3e (Harder et al. 2016) is the same as in the IFD protocol (Schrödinger Release 2020-2 2020). In this study, minimization of all residues’ atoms has been chosen as a sampling method to assess protein flexibility within a defined distance of 12 Å around the ligand. As an initial step for calculating the MM-GBSA binding energies of protein complexes, originating either from co-crystallized ligands or from IFD-output structures, the ligand has to be extracted as a separate entry for the use of this tool.

In general, MM-GBSA is an established method for rationalization of experimental findings (binding energies from co-crystallized ligands) and for improving the results of docking (Genheden and Ryde 2015). We used the following two parameters: (1) MM-GBSA dG Bind: The binding energy of the receptor and ligand as calculated by the Prime Energy, a Molecular Mechanics + Implicit Solvent Energy Function [kcals/mol] = PrimeEnergy (Optimized Complex) – PrimeEnergy (Optimized Free Ligand) – PrimeEnergy (Optimized Free Receptor); (2) MM-GBSA dG Bind (NS): A version of dG Bind that does not include contributions from receptor or ligand strain. [kcals/mol] = PrimeEnergy (Optimized Complex) – PrimeEnergy (Ligand Geometry From Optimized Complex) – PrimeEnergy (Receptor Geometry From Optimized Complex).


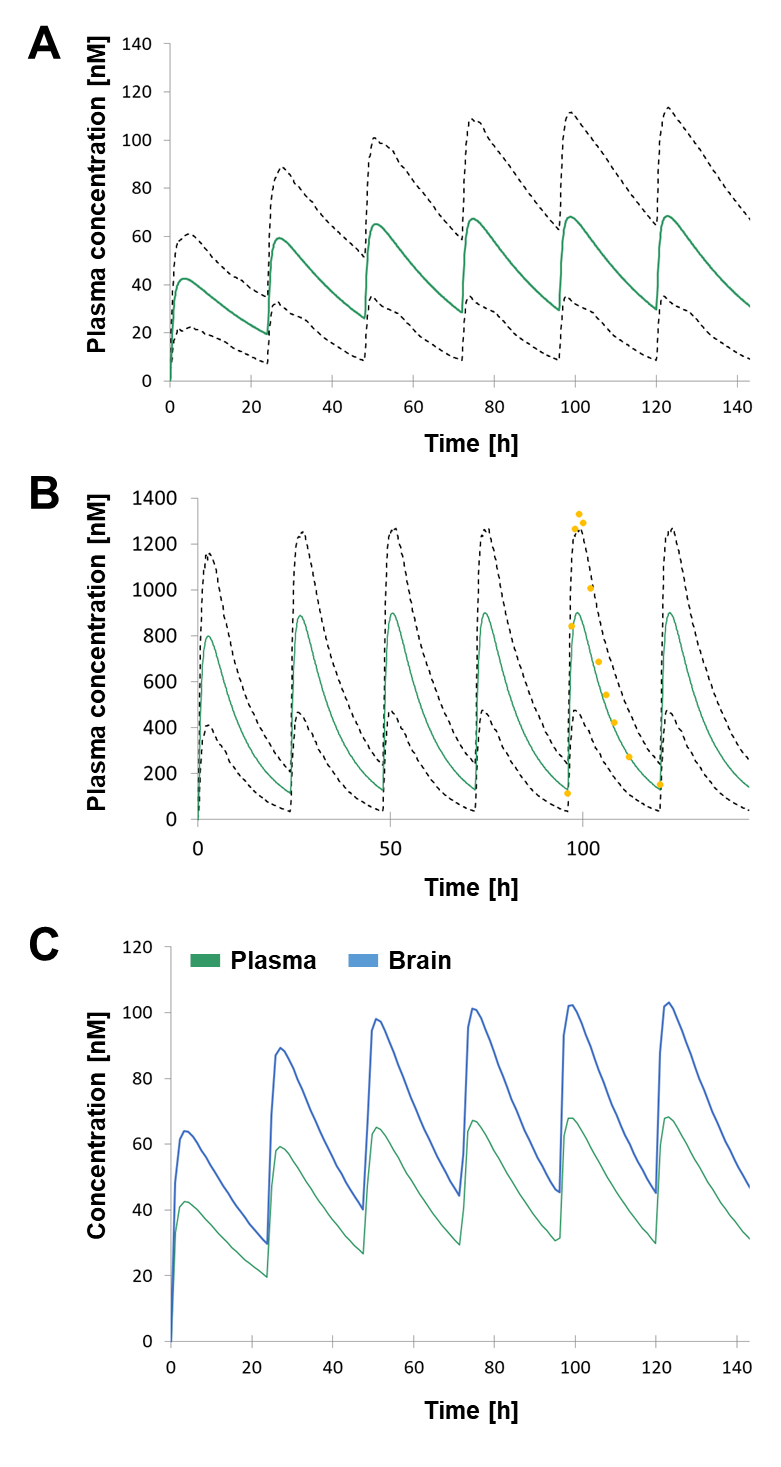


**Fig. S10: Physiologically-based toxicokinetic (PBTK) modeling of DN-IMI and atenolol in the human population.**

**(A)** The systemic exposure following an oral dose of 0.016 mg DN-IMI/kg body weight, given every 24 h intervals to 100 individuals (aged 20-50; 50% female) with randomly assigned phenotypic and genotypic properties typical for a caucasian Northern European population is shown. The predicted mean plasma concentrations of DN-IMI (green line) are shown with the 5^th^ and 95^th^ percentiles of the population (dashed lines). **(B)** The mean plasma concentrations of atenolol following multiple oral exposures of 50 mg every day for 6 days (green line) are shown with the 5^th^ and 95^th^ percentiles of the population (dashed lines). The population for the atenolol simulation matched the demographics of the subjects used in a clinical study (10 trials of 16 subjects aged 36-65; 31% female, (Andrawis et al. 2000)). The reported data are shown as yellow circles. The simulated mean atenolol C_max_ and AUC were 245 ng/mL and 2867 ng*h/ml, respectively, and are in reasonable agreement with the reported mean values (409.3 ng/mL for C_max_ and 3534 ng*h/ml for AUC). The simulated C_max_ and AUC were within 1.7 and 1.2 fold, respectively, of the observed values from this clinical study and were considered to be adequate for the purpose of this exercise. **(C)** The mean plasma (green) and brain (blue) concentrations of DN-IMI following multiple oral exposures of 0.016 mg DN-IMI/kg body weight, given every 24 h intervals to 100 individuals (aged 20-50; 50% female) with randomly assigned phenotypic and genotypic properties typical for a caucasian Northern European population are shown. The input parameters for DN-IMI and atenolol are given in table S8.

In clearance studies conducted with DN-IMI in a hepatocyte co-culture system, the measured in vitro intrinsic clearance was <0.143 ml/min/106 cells (i.e. below the limit of quantitation of the assay; in the same experiment the positive control compounds ketoprofen and prednisolone had measured intrinsic clearance values of 4.39 and 0.379, ml/min/106 cells, respectively (data not shown). As the rate of metabolism of DN-IMI was below the limit of quantitation of the clearance assay, no metabolic clearance was included in this DN-IMI PBTK model. This is a conservative assumption and metabolism components can be included in the model if data becomes available. Renal clearance for DN-IMI was assumed to be governed only by passive processes, with both filtration and passive reabsorption being accounted for. Passive permeability was scaled from the predicted permeability in the intestine (Peff 0.37 10-4 cm/s) accounting for the surface area of the proximal tubules in the kidney. The renal CL for atenolol also includes a component of active secretion and was entered into the model using reported transporter kinetic data (Yin et al. 2015). In the absence of equivalent data for DN-IMI, no active secretion component was included for this compound. If active secretion does occur for DN-IMI this would result in a lower exposure of DN-IMI in the body.

**Table S1: Compound list.**

| **Compound** | **Supplier** | **Order number** | **Function** | **Reference** |
| --- | --- | --- | --- | --- |
| ABT 594 | Sigma | Q53646 | neuronal non-α7 nACh receptor agonist | Donnelly-Roberts et al. 1998; Michelmore et al. 2002 |
| Acetylcholine chloride (ACh) | Sigma Aldrich | A6625 | Endogenous neurotransmitter |  |
| Advanced DMEM/F12 | Gibco | 12634010 |  |  |
| Boric acid | Sigma Aldrich | B7660 |  |  |
| Cal-520 AM | Biomol | ABD-21130 |  |  |
| Desnitro-Imidacloprid | Sigma Aldrich | 37052 | Metabolite of Imidacloprid |  |
| Dibutyryl cyclic-AMP sodium salt (cAMP) | Sigma Aldrich | D0627 |  |  |
| Dimethyl sulfoxide (DMSO) | Sigma Aldrich | D8418 |  |  |
| Recombinant human FGF basic (FGF-2) | R&D Systems | 4114-TC |  |  |
| Fibronectin | Sigma Aldrich | F1141 |  |  |
| Recombinant human GDNF protein (GDNF) | R&D Systems | 212-GD |  |  |
| L-glutamine solution | Sigma Aldrich | G7513 |  |  |
| Imidacloprid | Sigma Aldrich | 37894 | Neonicotinoid |  |
| Imidacloprid-olefin | Sigma Aldrich | 34534 | Metabolite of Imidacloprid |  |
| Mecamylamine | Sigma Aldrich | M9020 | nACh receptor antagonist | Papke et al. 2008; Capelli et al. 2011 |
| Methyllycaconitine | Sigma Aldrich | M168 | nACh receptor antagonist | Palma et al. 1996; Capelli et al. 2011 |
| MG 624 | Sigma Aldrich | M3184 | nACh receptor antagonist | Gotti et al. 2000; Capelli et al. 2011 |
| N2-supplement | Gibco | 17502048 |  |  |
| (-)-Nicotine | Sigma Aldrich | N3876 | non-selective nACh receptor agonist | Capelli et al. 2011 |
| PNU-120596 | Sigma Aldrich | P0043 | Positive allosteric nACh receptor modulator | Hurst et al. 2005; Grønlien et al. 2007; Williams et al. 2011 |
| Polyethyleneimine solution (PEI) | Sigma Aldrich | P3143 |  |  |
| Poly-L-ornithine (PLO) | Sigma Aldrich | P3655 |  |  |
| Sodium tetraborate | Sigma Aldrich | 221732 |  |  |
| Tetracycline hydrochloride | Sigma Aldrich | T7660 |  |  |
| Trypsin-EDTA (0.05 %) | Gibco | 25300062 |  |  |
| (+)-Tubocurarine chloride pentahydrate | Sigma | 93750 | non-selective nACh receptor antagonist | Chavez-Noriega et al. 2000; Jonsson et al. 2006 |

**Table S2: Overview of pEC_50_ values for agonist experiments.**

**Table S3: Overview of percentages of responsive cells of single-cell Ca^2+^-imaging.**

**Table S4: Overview of pIC_50_ values.**

**Table S5: Overview of pEC_50_ and maximum values for *Xenopus laevis* oocyte experiments.**

**Table S6: Overview of concentrations and technical replicates.**

(Table continued on next page.)

**Table S6: Overview of concentrations and technical replicates. (continued)**

(Table continued on next page.)

**Table S6: Overview of concentrations and technical replicates. (continued)**

**Table S7: IFD and MMG-BSA-energy calculation parameters.**


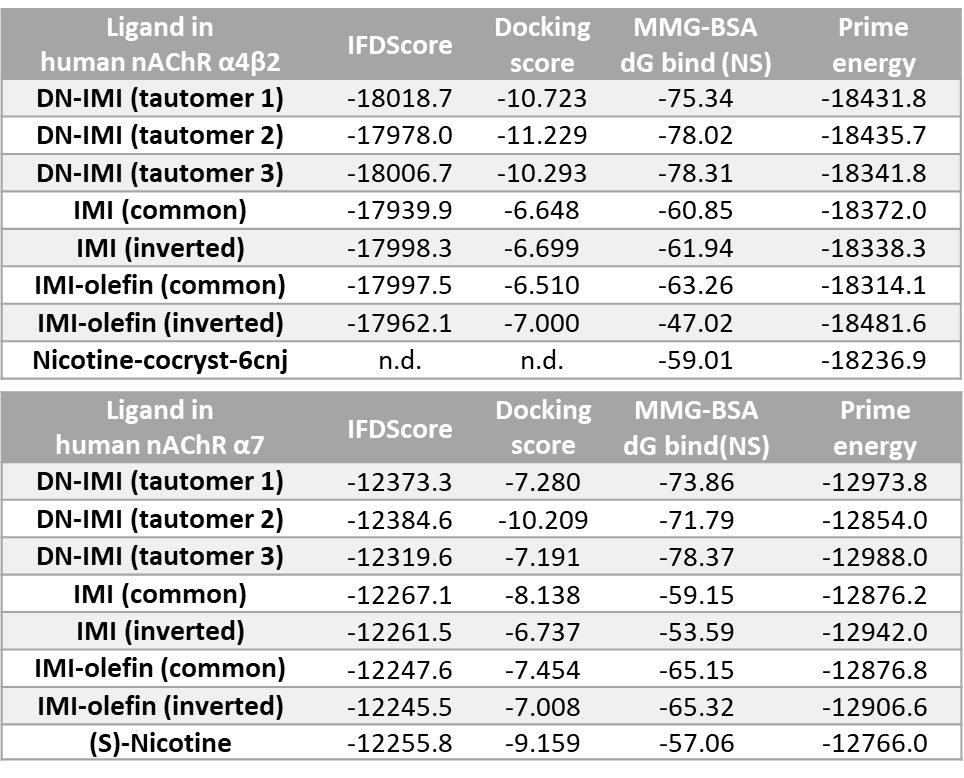


**Table S8: Input parameters used in the DN-IMI and atenolol PBTK model.**

| **Parameter** | **DN-IMI** | **Atenolol** | **Source** |
| --- | --- | --- | --- |
| Molecular weight (g/mol) | 210.7 | Pubchem | Pubchem |
| log P | 0.23 | 0.25 | DN-IMI average of 6 estimates from different sources. |
| Compound type | Monoprotic base | Monoprotic base |  |
| pKa | 9.6 | 9.6 | DN-IMI average of values in CHEMBL and ACD. Atenolol value taken from Dahlgren et al. (2016). |
| Blood/plasma ration [B/P] | 0.759 | 1.07 | DN-IMI was measured. Atenolol was taken from Taylor and Turner (1981). |
| Free fraction [fu] | 0.841 | 0.97 | DN-IMI was measured. Atenolol data was taken from Barber et al. (1978). |
| Main plasma binding protein | Human serum albumin | Human Serum albumin | Assumed. |
| **Absorption parameters** |  |  |  |
| Jejunal permeability  (10^-4^ cm/s) | 0.63 | 0.45 | Predicted – mechanistic permeability model (Sugano 2009). For atenolol the P_trans,0_ value was calibrated so that the jejunal permeability corresponded to the observed value in humans reported by Dahlgren et al. (2016). The same calibration to P_trans,0_ was applied to DN-IMI. |
| P_trans,0_ (10^-6^cm/s) | 120 | 120 |  |
| fu_gut_ | 1 | 1 |  |
|  |  |  |  |
| **Distribution Model** | **Full PBTK Model** | |  |
| V_SS_ (L/kg) | 0.85 | 0.88 | Predicted - Method 2 (Rodgers and Rowland 2007). Atenolol value was adjusted to be in line with the value reported by Dahlgren et al. (2016). |
| Metabolic Clint CYP3A4  (ml/min/pmol enzyme) | 0 | 0.00073 | Atenolol is 95% excreted into urine. |
| CL_R_ (L/h) | 3.2 | 9.64 | Predicted using Mech Kim model (Burt et al. 2016). Atenolol CL_R_ was adjusted to recover the mean CL_R_ reported after IV dosing by Kirch et al. (1981), Mason et al. (1979), and Wan et al. (1979). |

**Table S9: Benchmark responses (BMR) for the [Ca^2+^]_i_ responses of LUHMES neurons triggered by DN-IMI and IMI-olefin.**

Overview of the BMR10 and BMR20 concentrations (in log[M]) of the [Ca^2+^]_i_ response of LUHMES cells induced by DN-IMI and IMI-olefin. For each BMR, the benchmark concentrations (BMC) and their confidence interval (BMCL and BMCU) are shown; n.a.: BMR could not be determined due to low/no effect within the tested concentration range.

**Table S10: Benchmark responses (BMR) for the agonistic effects of nicotine and DN-IMI on several human nAChR subtypes expressed in *Xenopus laevis* oocytes.**

Overview of the BMR10 and BMR20 concentrations (in log[M]) of the inward current response induced by nicotine or DN-IMI on several human nAChR subtypes expressed in *Xenopus laevis* oocytes. For each BMR, the benchmark concentrations (BMC) and their confidence interval (BMCL and BMCU) are shown.

**Table S11: Benchmark responses (BMR) for the desensitizing effect of DN-IMI and IMI-olefin on nAChR signaling.**

Overview of the BMR10 and BMR20 concentrations (in log[M]) of the [Ca^2+^]_i_ response induced by nicotine, ACh, or ABT 594 after an initial stimulation of LUHMES cells with DN-IMI or IMI-olefin. For each BMR, the benchmark concentrations (BMC) and their confidence interval (BMCL and BMCU) are shown.

**References**

Andrawis NS, Battle MM, Klamerus KJ, et al (2000) A Pharmacokinetic and Pharmacodynamic Study of the Potential Drug Interaction between Tasosartan and Atenolol in Patients with Stage 1 and 2 Essential Hypertension. J Clin Pharmacol 40:231–241. https://doi.org/10.1177/00912700022008892

Barber HE, Hawksworth GM, Kitteringham NR, et al (1978) Protein binding of atenolol and propranolol to human serum albumin and in human plasma [proceedings]. Br J Clin Pharmacol 6:446P-447P

Burt HJ, Neuhoff S, Almond L, et al (2016) Metformin and cimetidine: Physiologically based pharmacokinetic modelling to investigate transporter mediated drug-drug interactions. Eur J Pharm Sci Off J Eur Fed Pharm Sci 88:70–82. https://doi.org/10.1016/j.ejps.2016.03.020

Capelli AM, Castelletti L, Chen YH, et al (2011) Stable expression and functional characterization of a human nicotinic acetylcholine receptor with α6β2 properties: discovery of selective antagonists. Br J Pharmacol 163:313–329. https://doi.org/10.1111/j.1476-5381.2011.01213.x

Chavez-Noriega LE, Gillespie A, Stauderman KA, et al (2000) Characterization of the recombinant human neuronal nicotinic acetylcholine receptors α3β2 and α4β2 stably expressed in HEK293 cells. Neuropharmacology 39:2543–2560. https://doi.org/10.1016/S0028-3908(00)00134-9

Dahlgren D, Roos C, Lundqvist A, et al (2016) Regional Intestinal Permeability of Three Model Drugs in Human. Mol Pharm 13:3013–3021. https://doi.org/10.1021/acs.molpharmaceut.6b00514

Donnelly-Roberts DL, Puttfarcken PS, Kuntzweiler TA, et al (1998) ABT-594 [(R)-5-(2-Azetidinylmethoxy)-2-Chloropyridine]: A Novel, Orally Effective Analgesic Acting via Neuronal Nicotinic Acetylcholine Receptors: I. In VitroCharacterization. J Pharmacol Exp Ther 285:777–786

Genheden S, Ryde U (2015) The MM/PBSA and MM/GBSA methods to estimate ligand-binding affinities. Expert Opin Drug Discov 10:449–461. https://doi.org/10.1517/17460441.2015.1032936

Gotti C, Carbonnelle E, Moretti M, et al (2000) Drugs selective for nicotinic receptor subtypes: a real possibility or a dream? Behav Brain Res 113:183–192. https://doi.org/10.1016/S0166-4328(00)00212-6

Grønlien JH, Håkerud M, Ween H, et al (2007) Distinct Profiles of α7 nAChR Positive Allosteric Modulation Revealed by Structurally Diverse Chemotypes. Mol Pharmacol 72:715–724. https://doi.org/10.1124/mol.107.035410

Harder E, Damm W, Maple J, et al (2016) OPLS3: A Force Field Providing Broad Coverage of Drug-like Small Molecules and Proteins. J Chem Theory Comput 12:281–296. https://doi.org/10.1021/acs.jctc.5b00864

Hurst RS, Hajós M, Raggenbass M, et al (2005) A Novel Positive Allosteric Modulator of the α7 Neuronal Nicotinic Acetylcholine Receptor: In Vitro and In Vivo Characterization. J Neurosci 25:4396–4405. https://doi.org/10.1523/JNEUROSCI.5269-04.2005

Ihara M, Okajima T, Yamashita A, et al (2008) Crystal structures of Lymnaea stagnalis AChBP in complex with neonicotinoid insecticides imidacloprid and clothianidin. Invert Neurosci 8:71–81. https://doi.org/10.1007/s10158-008-0069-3

Ihara M, Okajima T, Yamashita A, et al (2014) Studies on an Acetylcholine Binding Protein Identify a Basic Residue in Loop G on the β1 Strand as a New Structural Determinant of Neonicotinoid Actions. Mol Pharmacol 86:736–746. https://doi.org/10.1124/mol.114.094698

Jonsson M, Gurley D, Dabrowski M, et al (2006) Distinct Pharmacologic Properties of Neuromuscular Blocking Agents on Human Neuronal Nicotinic Acetylcholine Receptors: A Possible Explanation for the Train-of-four Fade. Anesthesiology 105:521–533

Karlin A (2002) Emerging structure of the Nicotinic Acetylcholine receptors. Nat Rev Neurosci 3:102–114. https://doi.org/10.1038/nrn731

Kirch W, Köhler H, Mutschler E, Schäfer M (1981) Pharmacokinetics of atenolol in relation to renal function. Eur J Clin Pharmacol 19:65–71. https://doi.org/10.1007/BF00558387

Li J, Abel R, Zhu K, et al (2011a) The VSGB 2.0 model: A next generation energy model for high resolution protein structure modeling. Proteins Struct Funct Bioinforma 79:2794–2812. https://doi.org/10.1002/prot.23106

Li S-X, Huang S, Bren N, et al (2011b) Ligand-binding domain of an α7-nicotinic receptor chimera and its complex with agonist. Nat Neurosci 14:1253–1259. https://doi.org/10.1038/nn.2908

Loser D, Hinojosa MG, Blum J, et al (2021) Functional alterations by a subgroup of neonicotinoid pesticides in human dopaminergic neurons. Arch Toxicol 95:2081–2107. https://doi.org/10.1007/s00204-021-03031-1

Madhavi Sastry G, Adzhigirey M, Day T, et al (2013) Protein and ligand preparation: parameters, protocols, and influence on virtual screening enrichments. J Comput Aided Mol Des 27:221–234. https://doi.org/10.1007/s10822-013-9644-8

Mason WD, Winer N, Kochak G, et al (1979) Kinetics and absolute bioavailability of atenolol. Clin Pharmacol Ther 25:408–415. https://doi.org/10.1002/cpt1979254408

Michelmore S, Croskery K, Nozulak J, et al (2002) Study of the calcium dynamics of the human α4β2, α3β4 and α1β1γδ nicotinic acetylcholine receptors. Naunyn Schmiedebergs Arch Pharmacol 366:235–245. https://doi.org/10.1007/s00210-002-0589-z

Morales-Perez CL, Noviello CM, Hibbs RE (2016) X-ray structure of the human α4β2 nicotinic receptor. Nature 538:411–415. https://doi.org/10.1038/nature19785

Ng HW, Leggett C, Sakkiah S, et al (2018) Competitive docking model for prediction of the human nicotinic acetylcholine receptor α7 binding of tobacco constituents. Oncotarget 9:16899–16916. https://doi.org/10.18632/oncotarget.24458

Noviello CM, Gharpure A, Mukhtasimova N, et al (2021) Structure and gating mechanism of the α7 nicotinic acetylcholine receptor. Cell 184:2121-2134.e13. https://doi.org/10.1016/j.cell.2021.02.049

Palma E, Bertrand S, Binzoni T, Bertrand D (1996) Neuronal nicotinic alpha 7 receptor expressed in Xenopus oocytes presents five putative binding sites for methyllycaconitine. J Physiol 491:151–161. https://doi.org/10.1113/jphysiol.1996.sp021203

Papke RL, Dwoskin LP, Crooks PA, et al (2008) Extending the analysis of nicotinic receptor antagonists with the study of α6 nicotinic receptor subunit chimeras. Neuropharmacology 54:1189–1200. https://doi.org/10.1016/j.neuropharm.2008.03.010

Rodgers T, Rowland M (2007) Rodgers T, Rowland M. 2006. Physiologically‐based Pharmacokinetic Modeling 2: Predicting the tissue distribution of acids, very weak bases, neutrals and zwitterions. J Pharm Sci 95:1238–1257. J Pharm Sci 96:3153–3154. https://doi.org/10.1002/jps.20857

Roos K, Wu C, Damm W, et al (2019) OPLS3e: Extending Force Field Coverage for Drug-Like Small Molecules. J Chem Theory Comput 15:1863–1874. https://doi.org/10.1021/acs.jctc.8b01026

Sakkiah S, Leggett C, Pan B, et al (2020) Development of a Nicotinic Acetylcholine Receptor nAChR α7 Binding Activity Prediction Model. J Chem Inf Model 60:2396–2404. https://doi.org/10.1021/acs.jcim.0c00139

Schrödinger Release 2020-2 (2020) Maestro. Schrödinger, LLC, New York, NY

Sugano K (2009) Theoretical investigation of passive intestinal membrane permeability using Monte Carlo method to generate drug-like molecule population. Int J Pharm 373:55–61. https://doi.org/10.1016/j.ijpharm.2009.02.002

Taylor EA, Turner P (1981) The distribution of propranolol, pindolol and atenolol between human erythrocytes and plasma. Br J Clin Pharmacol 12:543–548. https://doi.org/10.1111/j.1365-2125.1981.tb01263.x

Tomizawa M, Maltby D, Talley TT, et al (2008) Atypical nicotinic agonist bound conformations conferring subtype selectivity. Proc Natl Acad Sci 105:1728–1732. https://doi.org/10.1073/pnas.0711724105

Velisetty P, Chalamalasetti SV, Chakrapani S (2014) Structural Basis for Allosteric Coupling at the Membrane-Protein Interface in Gloeobacter violaceus Ligand-gated Ion Channel (GLIC) *. J Biol Chem 289:3013–3025. https://doi.org/10.1074/jbc.M113.523050

Walsh RM, Roh S-H, Gharpure A, et al (2018) Structural principles of distinct assemblies of the human α4β2 nicotinic receptor. Nature 557:261–265. https://doi.org/10.1038/s41586-018-0081-7

Wan SH, Koda RT, Maronde RF (1979) Pharmacokinetics, pharmacology of atenolol and effect of renal disease. Br J Clin Pharmacol 7:569–574. https://doi.org/10.1111/j.1365-2125.1979.tb04644.x

Williams DK, Wang J, Papke RL (2011) Investigation of the Molecular Mechanism of the α7 Nicotinic Acetylcholine Receptor Positive Allosteric Modulator PNU-120596 Provides Evidence for Two Distinct Desensitized States. Mol Pharmacol 80:1013–1032. https://doi.org/10.1124/mol.111.074302

Yin J, Duan H, Shirasaka Y, et al (2015) Atenolol Renal Secretion Is Mediated by Human Organic Cation Transporter 2 and Multidrug and Toxin Extrusion Proteins. Drug Metab Dispos Biol Fate Chem 43:1872–1881. https://doi.org/10.1124/dmd.115.066175
